# Insight into catalytic reduction of CO_2_ to methane with silanes using Brookhart's cationic Ir(iii) pincer complex†

**DOI:** 10.1039/c7ra13486j

**Published:** 2018-03-02

**Authors:** Shaoqin Fang, Hongcai Chen, Haiyan Wei

**Affiliations:** Jiangsu Key Laboratory of Biofunctional Materials, School of Chemistry and Materials Science, Jiangsu Provincial Key Laboratory for NSLSCS, Nanjing Normal University Nanjing 210097 China weihaiyan@njnu.edu.cn

## Abstract

Using density functional theory computations, we investigated in detail the underlying reaction mechanism and crucial intermediates present during the reduction of carbon dioxide to methane with silanes, catalyzed by the cationic Ir-pincer complex ((POCOP)Ir(H)(acetone)^+^, POCOP = 2,6-bis(dibutylphosphinito)phenyl). Our study postulates a plausible catalytic cycle, which involves four stages, by sequentially transferring silane hydrogen to the CO_2_ molecule to give silylformate, bis(silyl)acetal, methoxysilane and the final product, methane. The first stage of reducing carbon dioxide to silylformate is the rate-determining step in the overall conversion, which occurs *via* the direct dissociation of the silane Si–H bond to the C=O bond of a weakly coordinated Ir–CO_2_ moiety, with a free energy barrier of 29.5 kcal mol^−1^. The ionic S_N_2 outer-sphere pathway in which the CO_2_ molecule nucleophilically attacks at the η^1^-silane iridium complex to cleave the η^1^-Si–H bond, followed by the hydride transferring from iridium dihydride [(POCOP)IrH_2_] to the cation [O=C–OSiMe_3_]^+^, is a slightly less favorable pathway, with a free energy barrier of 33.0 kcal mol^−1^ in solvent. The subsequent three reducing steps follow similar pathways: the ionic S_N_2 outer-sphere process with silylformate, bis(silyl)acetal and methoxysilane substrates nucleophilically attacking the η^1^-silane iridium complex to give the ion pairs [(POCOP)IrH_2_] [HC(OSiMe_3_)_2_]^+^, [(POCOP)IrH_2_] [CH_2_(OSiMe_3_)_2_(SiMe_3_)]^+^, and [(POCOP)IrH_2_] [CH_3_O(SiMe_3_)_2_]^+^, respectively, followed by the hydride transfer process. The rate-limiting steps of the three reducing stages are calculated to possess free energy barriers of 12.2, 16.4 and 22.9 kcal mol^−1^, respectively. Furthermore, our study indicates that the natural iridium dihydride [(POCOP)IrH_2_] generated along the ionic S_N_2 outer-sphere pathway could greatly facilitate the silylation of CO_2_, with a potential energy barrier calculated at a low value of 16.7 kcal mol^−1^.

## Abstract 2

The transformation of CO_2_ and silanes to methane catalyzed by a cationic Ir–pincer complex is investigated and divided into four reducing steps. The first step is the rate-determining step of the overall catalytic cycle.

## Graphical abstract

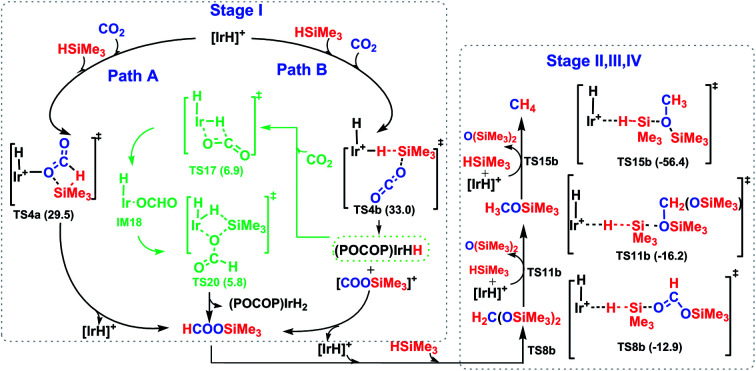

## Introduction

**Scheme 1:**
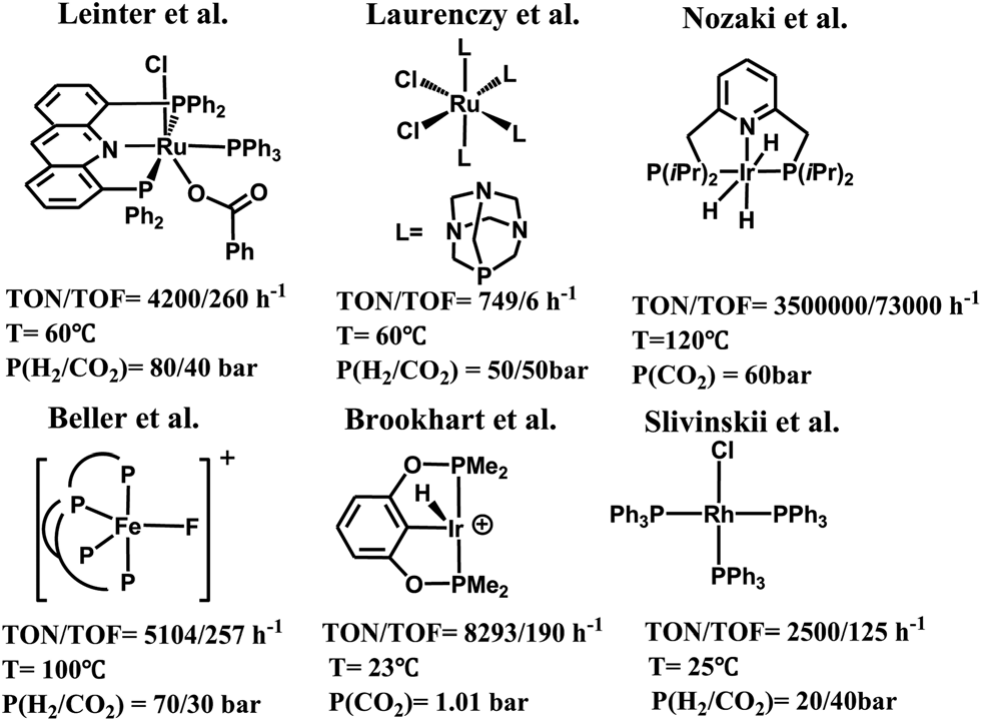
Examples of catalysts for the selective reduction of CO_2_ with H_2_ or silane catalyzed by ruthenium, iron or iridium-base complexes.

Carbon dioxide is a cheap, nontoxic and readily available carbon resource for the organic synthesis of valuable chemicals and materials.^1,2^ Transformation of carbon dioxide into fuels and useful organics such as formic acid,^3^ formaldehyde,^4^ methanol,^5^ and other derivatives^6^ is a topic of growing interest.^7,8^ Among others, significant efforts have been devoted to the chemical reduction of carbon dioxide using transition-metal catalysts.^9–14^ These catalysts have been extensively reviewed in the literature.^15^ Some representative examples are shown in Scheme 1, including a rhodium complex (RuCl_2_(PTA)_4_, PTA = 1,3,5-triaza-7-phosphaadamantane) reported by Laurenczy *et al.*, which catalyzed the hydrogenation of carbon dioxide affording formic acid as the only product,^16^ and an iron complex ([FeF(2)]BF_4_, 2 = tris(*o*-diphenylphosphinophenyl)-phosphine) reported by Beller *et al.*, which catalyzed the hydrogenation of carbon dioxide affording formates and formamides.^17^ Recently, a class of transition-metal catalysts supported by pincer ligands have been developed to achieve remarkable catalytic efficiency for carbon dioxide reduction. These include an Ir(iii) tri-hydride PNP-ligated complex, iPr(PNP)IrH_3_, reported by Nozaki *et al.*, which was used for the hydrogenation of carbon dioxide to formate,^18^ (PNP)RuH_2_CO reported by Pidko *et al.*,^19^ Ru(acriphos)(PPh_3_)(Cl)(PhCO_2_) reported by Leitner *et al.*,^20^ (POCOP)IrH_2_(MeCN) reported by Meyer,^21^ (PNHP)IrH_3_ (PNHP = HN{CH_2_CH_2_(P^i^Pr_2_)}_2_),^22^ and RhCl(PPh_3_)_3_,^23^*etc.*^24^ However, the use of hydrogen as the reducing agent to convert carbon dioxide generally requires higher pressures and/or temperatures and also involves the use of strong bases as co-reagents.^25^ In this regard, hydrosilane as a reductant has been explored as an alternative methodology, since the formation of the Si–O bond in silyl compounds is a thermodynamically favorable process.^26^ For example, carbon dioxide can be reduced to silyl formate, bis(silyl)acetal or silylether under the catalysis of [ReHBr(NO)(PR_3_)_2_]/B(C_6_F_5_)_3_,^27^ [Cp*_2_Sc][HB(C_6_F_5_)_3_]1_CIP_,^28^*cis*/*trans*-[RuCl_2_(MeCN)_4_],^29^ (BDP)CuH,^30^ and other Rh, Ir, Ru, Cu and Fe complexes,^31^ and main group catalysts including the frustrated Lewis acid B(C_6_F_5_)_3_,^32^ and N-heterocyclic carbenes.^33^ Notably, the Brookhart group presented a cationic Ir-pincer complex, (POCOP)Ir(H)(acetone)^+^ (POCOP = 2,6-bis(dibutylphosphinito)phenyl), to catalyze the hydrosilylation of carbon dioxide under mild reaction conditions, and achieved a high turnover number of 8300 and moderate turnover frequency (660/h at 60 °C).^34^ It is worth noting that by using this Ir-pincer catalyst, carbon dioxide could even be reduced to methane in high yields with less sterically hindered silanes.

**Scheme 2:**
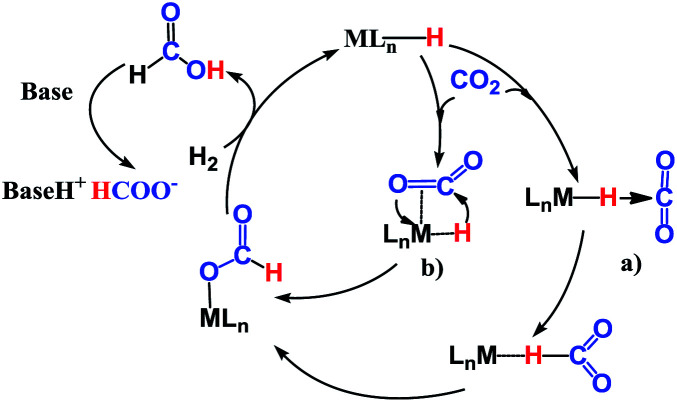
General reaction mechanism for transition-metal complexes catalyzing the hydrogenation of CO_2_ to formic acid or formate.

**Scheme 3:**
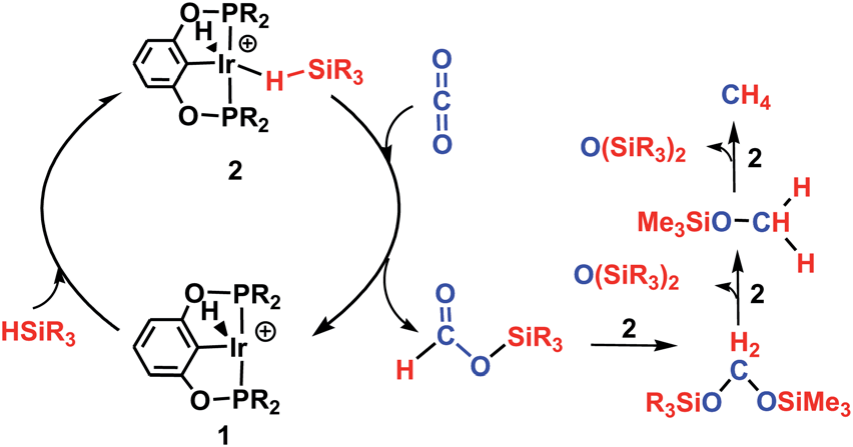
Schematic representation of the hydrosilylation of CO_2_ to methane using the cationic Ir(iii)-pincer complex proposed by Brookhart *et al.*

The reaction mechanism for carbon dioxide transformation mediated by transition-metal complexes has been studied extensively.^35^ As exemplified by Scheme 2, the common feature is the insertion of carbon dioxide into the metal–hydrogen bond of a metal hydride. Two general pathways have been identified: (a) *via* transfer of the hydride directly from the metal complex; and (b) *via* prior coordination of carbon dioxide to the metal center, followed by carbon dioxide abstracting a hydride from the metal center. Both pathways reduce carbon dioxide to a formate ion (HCOO^−^) around the metal center, forming a metal formate complex. Then, the formate ion or its derivative is eliminated as the metal formate intermediate reacts with H_2_. The hydrogenation of carbon dioxide to formic acid *via* these two modes, especially that involving insertion into the M–H bond of a metal complex, is considered to be the first elemental step in transition-metal-catalyzed hydrogenation/hydrosilylation reactions, which have been the subject of several reviews.^36–38^ The reaction mechanisms mediated by transition-metal complexes are usually complex. For the hydrosilylation of carbon dioxide into methane catalyzed by the cationic Ir-pincer complex, Brookhart and co-workers postulated an unconventional pathway. As shown in Scheme 3, the reaction occurs through activation of the Si–H bond of hydrosilane by the electrophilic Ir(iii) ion, forming a silane–iridium adduct as the step initiating the catalytic cycle. The silane–iridium adduct acts as an effective catalyst to reduce carbon dioxide to a silylformate (HCOOSiR_3_) product. Then, the silylformate substrate reacts with the silane–iridium adduct to provide bis(silyl)acetal (R_3_SiOCH_2_OSiR_3_), methoxysilane (R_3_SiOCH_3_) intermediates and finally, methane (CH_4_). This mechanistic proposal is remarkable and represents a new way of activating carbon dioxide using transition-metal complexes. The corresponding catalytic cycle is an outer-sphere mode, in which insertion of a carbon dioxide molecule into the metal-hydride bond does not occur.^39^ However, the detailed underlying reaction mechanism for the complete reduction of carbon dioxide to methane by the cationic Ir-pincer complex has not been investigated computationally, and the reaction mechanism is not yet understood in detail. Indeed, few examples of transition-metal catalyst systems are known to be active for the selective reduction of CO_2_ to methane.^40^ To better understand the reduction of CO_2_ mediated by the cationic Ir-pincer complex with silanes, we sought to explore the mechanism in more detail by employing DFT calculations. Our purpose was to uncover characteristic features of the electronic process of each elementary step during the catalytic reaction. An in-depth density functional theory (DFT) study of this system allows for further development on the conversion of carbon dioxide under the catalysis of transition-metal complexes.

## Computational methodology

All molecular geometries of the model complexes were optimized at the DFT Becke3LYP (B3LYP)^41^ level and by using the hybrid meta exchange–correlation M06 functional,^42^ which includes a medium-range correlation as implemented in Gaussian 09.^43^ The effective core potentials (ECPs) of Hay and Wadt with double-*ζ* valence basis sets (LanL2DZ)^44^ were used to describe the Ir atom. In addition, polarization functions were added for Ir (*ζ*_f_ = 0.938).^45^ The 6-311g(d,p) basis set was used for all other atoms, including C, H, P, Si and O. Frequency calculations at the same level of theory were performed to verify all stationary points as minima (zero imaginary frequency) and transition states (one imaginary frequency), as well as to provide free energies at 298.15 K, including entropic contributions. All transition states were verified to connect the respective minima through optimizations following initial intrinsic reaction coordinate calculations. To obtain the relative solvation-free energies, we used a continuum medium to perform single-point calculations for all the species under study using the SMD solvation model (an IEFPCM calculation with radii and non-electrostatic terms for Truhlar and coworkers' SMD solvation model),^46^ as implemented in Gaussian 09. CH_2_Cl_2_ was used as the solvent.

A model catalyst was used where the large butyl/isopropyl substituents at the carbon atom in the tridentate POCOP-pincer ligand were replaced with methyl groups. Trimethylsilane was used as a model silane. The final Gibbs energies (Δ*G*) reported in this article are based on B3LYP energies with Gibbs energy corrections (at 298.15 K), solvation corrections, and corrections for dispersion effects using the method of Grimme.^47^ Furthermore, it should be noted that the entropic contribution in a solvent medium is overestimated for a reaction using the ideal gas phase model. To reduce the overestimation of the entropy contribution in the results, we adopted the approximate approach proposed by Martin *et al.*,^53^*i.e.*, a reaction from *m*- to *n*-components has an additional correction of (*n* − *m*) × 4.3 kcal mol^−1^. Detailed comparisons of the different functionals (B3LYP, B3LYP-D and M06) are listed in the ESI.† The geometries are displayed using CYLview.^48^

## Results and discussion

### Overall catalytic mechanism

The following part of the paper is devoted to our theoretical analysis of the cationic Ir-pincer complex catalyzing the hydrosilylation of CO_2_, following a stepwise process comprising four steps. First, CO_2_ is reduced to give silylformate (HCOOSiMe_3_). Then, the silylformate is reduced to give bis(silyl)acetal (H_2_C(OSiMe3)_2_), methoxysilane (H_3_COSiMe_3_) and finally, methane (CH_4_).

#### Stage I: hydrosilylation of CO_2_ to silylformate (HCOOSiMe_3_)

Three different pathways were explored for the hydrosilylation of CO_2_ to silylformate under the catalysis of the cationic Ir-pincer complex: (a) the cationic Ir-pincer complex activating CO_2_ first, followed by the Ir–CO_2_ moiety activating a free silane molecule; (b) the cationic Ir-pincer complex activating the silane first as proposed by Brookhart, by coordination of Me_3_SiH to the iridium atom, followed by the silane–iridium adduct activating a CO_2_ molecule; and (c) CO_2_ inserting into the iridium-hydride bond of the cationic Ir-pincer complex, generating iridium formate, and reacting with a free silane to generate silylformate.

**Fig. 1:**
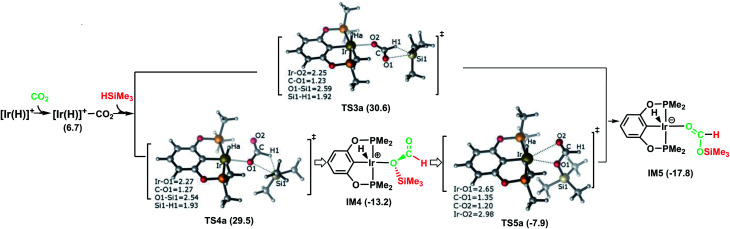
Schematic representation of stage I of the reduction of CO_2_ to silylformate (HCOOSiMe_3_) *via* pathway A (dissociation of free silanes to weakly coordinate CO_2_). The bond distances are shown in Å.

In Fig. 1, the reaction pathway representing the Ir–CO_2_ moiety to activate a free silane is illustrated, together with the optimized structures of key stationary points that were located. Carbon dioxide exhibits poor ligand properties toward the cationic Ir-pincer complex. An η^1^_O_-coordinated Ir–CO_2_ complex is located as CO_2_ weakly coordinates to the iridium atom, *d*(Ir⋯O(CO_2_)) = 2.43 Å. The adduct formation is endothermic by +6.7 kcal mol^−1^. Subsequently, dissociation of a free silane Si–H bond to the C=O bond of the weakly coordinated carbon dioxide would directly generate silylformate. Here, two metathesis transition states were located: TS3a and TS4a. In these two four-membered-ring transition states, the Si and H atoms of free silanes are approaching the C=O bonds of CO_2_, respectively, as shown in Fig. 1. The Si–H bond breaks (*d*(Si1⋯H1) = 1.92 (1.93 Å, TS4a)), new Si–O bonds begin to form (*d*(Si1⋯O1) = 2.59 (2.54 Å, TS4a)) and new H–C bonds begin to form (*d*(C⋯H1) = 1.23 (1.27 Å, TS4a)). The Ir–O(CO_2_) bonds become shortened to 2.25 and 2.27 Å, respectively. The O–C–O bond angles of CO_2_ are reduced significantly to 136° and 134°, respectively. The two transition states show only a small energetic difference, with energies of 30.6 kcal mol^−1^ and 29.5 kcal mol^−1^ in solvent. Both transition states yield the O-bridged silylformate iridium intermediates (IM4 and IM5). Nevertheless, IM4 can isomerize to the more stable intermediate IM5 by crossing a low barrier of 5.3 kcal mol^−1^ (TS5a relative to IM4). TS5a represents an η^2^_O,O_-carbonyl transition state, with Ir⋯O bond distances of 2.65 and 2.98 Å, respectively. Because the two silylformate iridium intermediates can be readily interconverted, only the more stable intermediate IM5 is considered in the subsequent studies. Therefore, TS4a is the highest stationary point along pathway A, wherein the Ir–CO_2_ moiety activates free silanes and generates the silylformate iridium complex, whose energy is 29.5 kcal mol^−1^ lower than that of the reactants ([Ir(H)]^+^ + silane + CO_2_). The reaction is exergonic by −17.8 kcal mol^−1^.

**Fig. 2:**
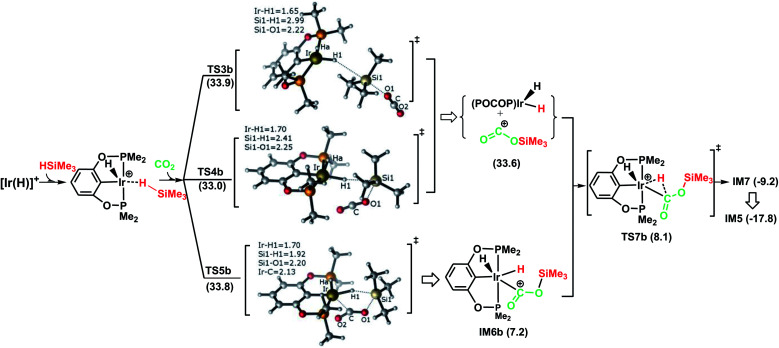
Schematic representation of stage I of the reduction of CO_2_ to silylformate (HCOOSiMe_3_) *via* pathway B (the ionic outer-sphere mechanism). The bond distances are shown in Å.

Fig. 2 illustrates the pathway for the silane–iridium adduct activating carbon dioxide, together with the energetic results and the optimized structures of key stationary points. This pathway starts with silane η^1^-binding to the iridium atom of the cationic Ir-pincer complex, generating an η^1^-silane iridium complex IM2 (see Table S1 in the ESI† for a comparison of the key structures, with an X-ray single crystal structure of the complex with trimethylsilane coordinated end-on to the iridium center, as reported by Brookhart^49^). The interaction between iridium and silane activates the Si–H bond, as shown by the elongated Si–H bond distance (1.24 *vs.* 1.16 Å in free silane), and results in a net charge of 1.584*e* on the silicon atom as compared to 1.345*e* in free silane, thus making it favorable for CO_2_ molecules to attack. Specifically, it is worth noting that the silane–iridium complexes have been suggested to be active in the reduction of a series of organic substrates, including alkyl halides, carbonyl compounds and amines, *etc.*^50^ The underlying reduction reaction mechanism has also been computed recently by several groups, and characterized as the ionic outer-sphere mechanism.^51^ After a thorough investigation into the possibilities of how the silane–iridium complex reduces carbon dioxide, we were able to locate three transition states. In the first transition state, TS3b (Fig. 2), a carbon dioxide molecule attacks the silicon atom at the face opposite to the iridium atom. TS3b can be viewed as a very late S_N_2-Si transition state, in which the silicon atom has a distorted trigonal bipyramidal structure. The Si–H bond is broken and becomes significantly elongated to be 2.99 Å, while Ir–H and Si–O bonds become fully formed (1.65 Å and 2.22 Å, respectively). The four atoms of O–Si⋯H–Ir maintain their linear arrangement (179°). The second transition state, TS4b, involves CO_2_ nucleophilically attacking the silicon atom of the η^1^-silane iridium complex from the same side as the iridium atom. At this transition state (Fig. 2), the Si–H bond becomes longer to 2.41 Å, while the Ir–H and Si–O distances become shorter (*d*(Si1⋯O1) = 2.25 Å, *d*(Ir⋯H1) = 1.70 Å), indicating that the silane Si–H bond is almost broken and the Ir–H and Si–O bonds are almost formed. The O–C–O bond angle stays linear at 175°, while the four atoms of O–Si⋯H–Ir are most perpendicular, at 68°. Furthermore, we located a nontraditional ionic outer-sphere transition state, TS5b, where a carbon dioxide molecule also attacks the silicon atom of the η^1^-silane iridium complex at the same side as the iridium atom. However, at TS5b, accompanying the cleavage of the Si–H bond, a silane hydrogen atom is transferred to the iridium metal (*d*(Ir–H) = 1.70 Å), while the silyl moiety transfers to CO_2_ (*d*(Si–O) = 2.20 Å). Simultaneously, CO_2_ bonds with the iridium atom with an Ir–C distance of 2.13 Å. Another interesting aspect of this transition state is the O–C–O bond angle of CO_2_, which is reduced to 141°. All three ionic outer-sphere transition states (TS3b, TS4b and TS5b) result in a concerted transfer of SiMe_3_^*δ*+^ moieties to CO_2_ and H^*δ*−^ moieties to the iridium atom, to give an ion pair comprising iridium dihydride [IrH_2_(POCOP)] and the cation [O=C–OSiMe_3_]^+^. A structural reorganization in the ion pair gives IM6b, with the cation [O=C–OSiMe_3_]^+^ binding to iridium dihydride through the carbon atom. From IM6b, the iridium dihydride transfers a hydride to the carbon atom of [O=C–OSiMe_3_]^+^ through the transition state TS7b, affording a H-bound silylformate iridium complex (IM7). The hydride transfer process represents a very small barrier, of a negligible 0.9 kcal mol^−1^ (TS7b relative to IM6b). As expected, the rate-determining step for the η^1^-silane iridium complex to activate carbon dioxide, generating the intermediate of silylformate, is the ionic S_N_2 outer-sphere transition states. Interestingly, we found three ionic S_N_2 transition states possessing similar activation energies: 33.9 kcal mol^−1^ (TS3b), 33.0 kcal mol^−1^ (TS4b) and 33.8 kcal mol^−1^ (TS5b) relative to the reactants ([Ir(H)]^+^+ silane + CO_2_).

**Fig. 3:**
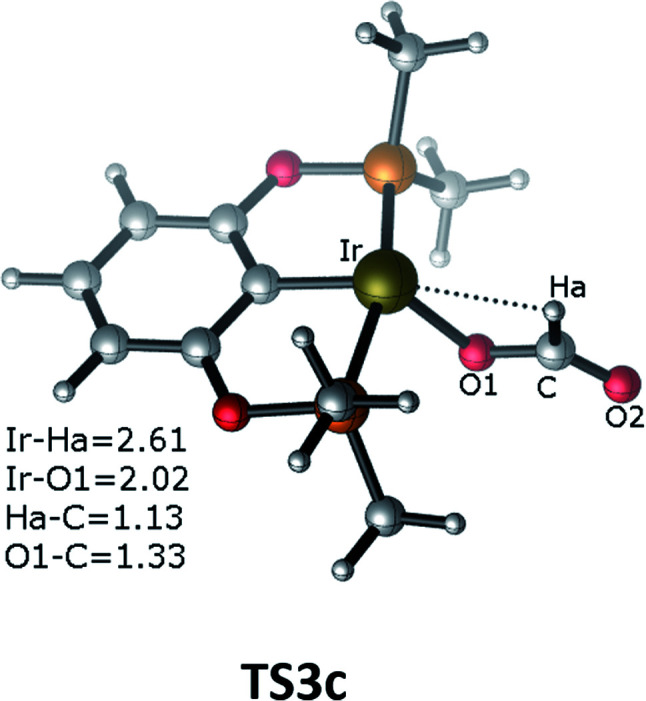
Optimized geometries of the transition state TS3c. The bond distances are shown in Å.

The third pathway illustrates carbon dioxide insertion into the Ir–H bond of the cationic Ir-pincer complex. Insertion of carbon dioxide (and other unsaturated organic substrates) into the M–H bond of a metal complex has been suggested as being the key step in catalytic hydrogenation.^52^ The insertion step is identified as a four-membered-ring transition state (TS3c), where the Ir–H bond is broken (*d*(Ir⋯Ha) = 2.61 Å), and the C–H bond is correspondingly formed (*d*(C⋯Ha) = 1.13 Å). It is worth noting that the apical H atom around the iridium atom deviates considerably from the apical position and is largely elongated when approaching the carbon atom of CO_2_ in the transition state TS3c. The barrier for TS3c is calculated to be significantly high, at 44.5 kcal mol^−1^ (TS3c relative to the reactants ([Ir(H)]^+^ + silane + CO_2_). The following steps along pathway C are shown in the ESI (Fig. S3†) (Fig. 3).

**Fig. 4:**
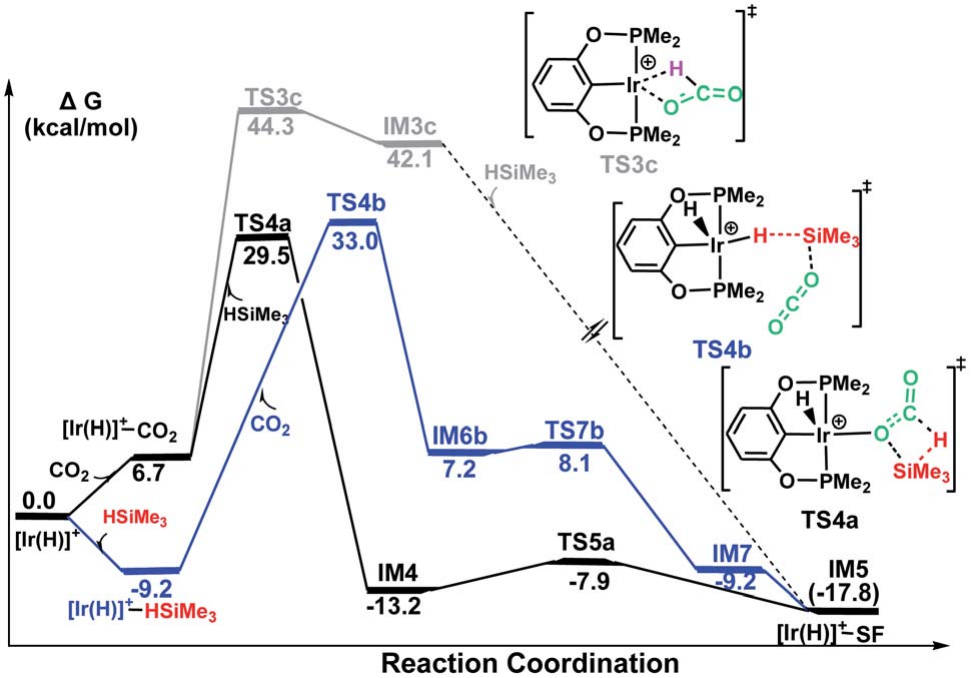
Summary of the results from the calculations on stage I of the hydrosilylation of CO_2_ to silylformate (HCOOSiMe_3_, SF) under the catalysis of [Ir(H)]^+^*via* three possible pathways: pathway A (black, silane Si–H bond dissociation to C=O bond of the weakly coordinated CO_2_), pathway B (blue, the ionic outer-sphere mechanistic pathway) and pathway C (gray, insertion of CO_2_ into the Ir–H bond). The solvent-phase Gibbs free energies [Δ*G*(sol)] are in kcal mol^−1^.

The calculated free energies of three pathways calculated for the cationic Ir-pincer complex catalyzing the hydrosilylation of CO_2_ to yield silylformate (HCOOSiMe_3_) are compared and shown in Fig. 4. Our DFT energetic results indicate that the insertion pathway can be ruled out, because the free energy barrier is as high as 44.3 kcal mol^−1^ and is ∼10 kcal mol^−1^ higher than the other two pathways. Therefore, a carbon dioxide molecule cannot directly insert into the Ir–H bond of the cationic Ir-pincer complex. However, it was found that the energy barrier for the ionic S_N_2 outer-sphere pathway (TS4b, 33.0 kcal mol^−1^, the highest energy along pathway B) is 3.5 kcal mol^−1^ higher than the dissociation pathway A (TS4a, 29.5 kcal mol^−1^, the highest energy along pathway A) in the solvent. When comparing the gas-phase electronic energies, TS4a was calculated to be 1.6 kcal mol^−1^ higher than TS4b. Nevertheless, considering the small energy difference of 3.5 kcal mol^−1^ in solvent and the inaccuracy of the computational method, we speculate that both pathways of A and B could be operating under the working conditions, with pathway A being slightly more favorable.

#### Stage II: hydrosilylation of silylformate (HCOOSiMe_3_) to bis(silyl)acetal (H_2_C(OSiMe3)_2_) or formaldehyde (H_2_C=O)

**Fig. 5:**
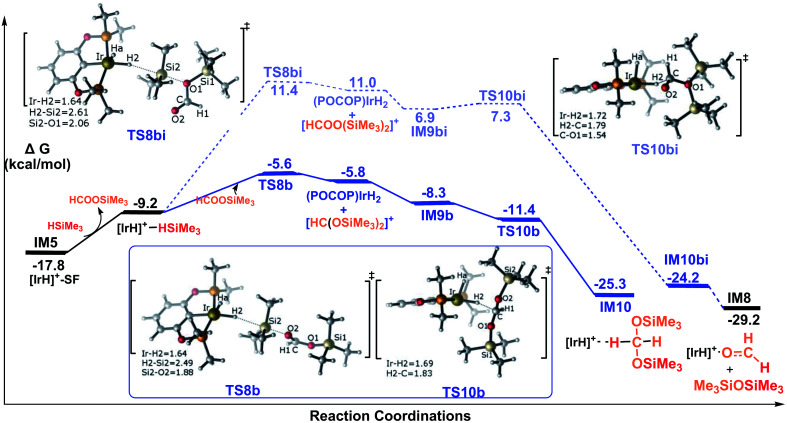
Summary of the results from the calculations for stage II of the hydrosilylation of silylformate to bis(silyl)acetal or formaldehyde catalyzed by the cationic Ir-pincer complex *via* pathway B. The solvent-phase Gibbs free energies are in kcal mol^−1^.

Three similar possible pathways for the cationic Ir-pincer complex catalyzing the hydrosilylation of silylformate substrate were studied. Pathway A, involving dissociation of a second silane Si–H bond to the C–O bond of silylformate, and pathway C, involving silylformate insertion into the Ir–H bond, were calculated to have higher activation free energies of 38.7 kcal mol^−1^ (TS6a) and 34.1 kcal mol^−1^ (TS7c), respectively (see Fig. S2 in the ESI† for more details about the attack model). Therefore, only the free energy changes of the favorable pathway B leading to bis(silyl)acetal or formaldehyde, as shown in Fig. 5, are discussed.

Pathway B corresponds to the ionic S_N_2 outer-sphere mechanism with the silylformate nucleophilically attacking the η^1^-silane iridium complex. Initially, silylformate is liberated from the iridium atom. For the next step, we considered two attacking models. We first considered an activation mode with the exposed oxygen atom of the C=O bond of silylformate acting as the entering group to attack the silicon atom of the η^1^-H–Si bond (Fig. 5). The corresponding transition state featuring the ionic S_N_2 outer-sphere process was identified as TS8b, in which the Si2–H2 bond is elongated to 2.49 Å, and the O–Si2 and Ir–H2 bonds are shortened to 1.88 Å and 1.64 Å, respectively. Alternatively, the exposed oxygen atom of the C–O(SiMe_3_) bond of the silylformate substrate could also act as the entering group to attack the Si center of the η^1^-H–Si bond. The transition state was identified as TS8bi. At this transition state, accompanying the Si2–H2 bond heterolytic cleavage, the silyl moiety and the silane hydrogen binds to silylformate and iridium, respectively (*d*(Si2⋯O1) = 2.06 Å, *d*(Ir⋯H2) = 1.64 Å). Both of the S_N_2 transition states yield ionic pair intermediates. TS8b leads to an ion pair comprising iridium dihydride [IrH_2_(POCOP)] and the cation [HC(OSiMe_3_)_2_]^+^. Alternatively, the ionic pair of iridium dihydride [IrH_2_(POCOP)] and the cation [HCOO(SiMe_3_)_2_]^+^ are formed from TS8bi. At the next step, the weakly bound moiety in the ion pairs can reorganize, followed by hydride transfer to give the corresponding product. The hydride on the iridium dihydride transfers to the cation [HC(OSiMe_3_)_2_]^+^ to give a bis(silyl)acetal–Ir adduct, passing TS10b. Alternatively, the hydride on the iridium dihydride migrates to the carbon atom of the cation [HCOO(SiMe_3_)_2_]^+^ passing TS10bi. Through TS10bi, disiloxane O(SiMe_3_)_2_ can easily dissociate to give the Ir-bound formaldehyde adduct. Our DFT results show that both hydride transfer processes are thermodynamically favorable and proceed effectively with a negligibly barrier. TS10b is barrier free. TS10bi was computed to be 0.4 kcal mol^−1^ relative to the ionic pair of [IrH_2_(POCOP)] and the cation [HCOO(SiMe_3_)_2_]^+^ in solvent.

Therefore, the rate-determining steps along the ionic S_N_2 outer-sphere pathway are the transition states TS8b and TS8bi. The energy needed for the C=O bond of silylformate to attack the η^1^-H–Si bond is 12.2 kcal mol^−1^ (TS8b relative to the silylformate iridium complex IM5), which is 17.0 kcal mol^−1^ lower than that needed for the C–O(SiMe_3_) bond of silylformate to attack the η^1^-H–Si bond (29.2 kcal mol^−1^, TS8bi relative to the silylformate iridium complex IM5). Therefore, the ionic S_N_2 outer-sphere pathway leading to the bis(silyl)acetal–Ir adduct is greatly favored relative to the pathway leading to the formaldehyde–Ir adduct. In other words, nucleophilic attack of the C=O double bond is highly favorable, while the nucleophilic attack of the C–O(SiMe_3_) single bond is highly unlikely. This argument finds support from the structural characteristics calculated for the two transition states. As shown in the optimized structures of TS8b and TS8bi, the Si2–O2 bond distance is 2.49 Å in TS8b, which is shorter than the Si2–O1 bond distance of 2.61 Å in TS8bi. This indicates a stronger interaction between the oxygen atom of the C=O bond in silylformate and the silicon atom of η^1^-silane, compared to that between the oxygen atom of C–O(SiMe_3_) in silylformate and the silicon atom of η^1^-silane.

In summary, as shown in Fig. 5, we conclude that the hydrosilylation of silylformate catalyzed by the cationic iridium complex is an exothermic process with a value of −7.5 kcal mol^−1^ (IM5 to IM10). The most favorable pathway to generate the bis(silyl)acetal substrate is *via* the ionic S_N_2 outer-sphere pathway, with an activation free energy barrier of only 12.2 kcal mol^−1^ (TS8b relative to IM5). The generation of formaldehyde was found to be less energetically favorable, associated with an activation free energy of 29.2 kcal mol^−1^ (TS8bi relative to IM5). Thus, the generation of formaldehyde is prevented from participating in the reaction. Our observation suggests that the bis(silyl)acetal substrate, but not the formaldehyde product, is observed in the hydrosilylation of CO_2_ catalyzed by the cationic Ir-pincer complex, which is consistent with the experimental results obtained by Brookhart *et al.*^34^

#### Third and fourth reduction steps: reducing bis(silyl)acetal (H_2_C(OSiMe_3_)_2_) to methoxysilane (H_3_COSiMe_3_) and reducing methoxysilane (H_3_COSiMe_3_) to methane (CH_4_)

**Fig. 6:**
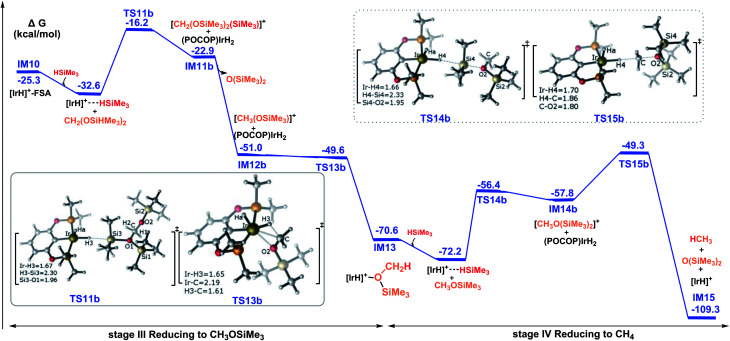
Summary of the results for the hydrosilylation of bis(silyl)acetal to methoxysilane and methane, catalyzed by the cationic iridium complex *via* the ionic outer-sphere mechanistic pathways. The solvent-phase Gibbs free energies [Δ*G*(sol)] are in kcal mol^−1^.

In the continuous reduction of bis(silyl)acetal to methoxysilane and then to methane, mediated by the cationic Ir-pincer complex, the favorable pathway takes place *via* two sequential ionic S_N_2 outer-sphere processes. Fig. 6 shows the energy profiles for the two steps. When the bis(silyl)acetal substrate nucleophilically attacks the η^1^-silane iridium complex, the ionic S_N_2 transition state TS11b can be identified. At TS11b, a new Ir–H3 bond is partially formed (1.67 Å), accompanied by the significant elongation of the Si3–H3 bond (2.30 Å). Together with silyl binding to bis(silyl)acetal and a silane hydrogen binding to the iridium atom, an ion pair comprising iridium dihydride and the cation [CH_2_(OSiMe_3_)_2_(SiMe_3_)]^+^ is formed. Next, iridium dihydride transfers a hydride to the carbon atom of the cation [CH_2_(OSiMe_3_)_2_(SiMe_3_)]^+^*via* the transition state TS13b, giving a methoxysilane–Ir adduct. The rate-determining step is calculated to be associated with an activation barrier of 16.4 kcal mol^−1^ (TS11b relative to the η^1^-silane iridium complex and bis(silyl)acetal).

Then, the reduction of methoxysilane *via* the ionic S_N_2 outer-sphere reaction pathway leads to the final product methane. The ionic S_N_2 outer-sphere transition state was identified as TS14b. At TS14b, the new Ir–H4 bond is partially formed (1.66 Å), accompanied by the significant elongation of the Si4–H4 bond (2.33 Å). TS14b gives the ion pair comprising iridium dihydride and the cation [CH_3_O(SiMe_3_)_2_]^+^. Subsequently, iridium dihydride transfers a hydride to the carbon of the cation [CH_2_(OSiMe_3_)_2_(SiMe_3_)]^+^, leading to the formation of methane, by passing TS15b. The rate-determining step along the ionic S_N_2 outer-sphere pathway for the reduction of methoxysilane to methane was calculated to have an activation barrier of 22.9 kcal mol^−1^ (TS15 relative to the η^1^-silane iridium complex and methoxysilane). Therefore, the third stage of the reduction reaction of bis(silyl)acetal to methoxysilane along TS11b → IM11b → IM12b → TS13b → IM13 and the fourth stage of the reduction of methoxysilane to methane along TS14b → IM14b → TS15b → IM15 have low activation barriers of 16.4 kcal mol^−1^ and 22.9 kcal mol^−1^, respectively.

#### The iridium dihydride complex [IrH_2_(POCOP)] catalyzing the reduction of CO_2_ with silanes

**Fig. 7:**
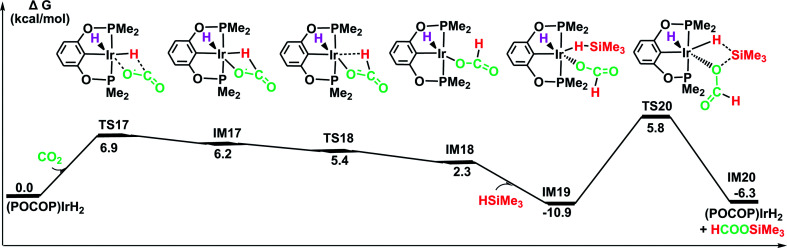
The DFT results for the iridium dihydride complex [IrH_2_(POCOP)] catalyzing the hydrosilylation of CO_2_. Optimized geometries of key stationary points are displayed in Fig. S11.†

Our DFT results show that the iridium dihydride can be generated *in situ* along the ionic S_N_2 outer-sphere pathways for the hydrosilylation of carbon dioxide catalyzed by the cationic Ir-pincer complex, as detailed in Fig. 4–6. Since metal dihydrides have previously been explored to be effective catalysts in the conversion of carbon dioxide,^54^ we investigated the likelihood of the iridium dihydride-catalyzed reduction of CO_2_ with silanes. The optimized structures of the relevant mechanism and relative free energy profiles are depicted in Fig. 7. According to our calculations, carbon dioxide insertion into the Ir–H bond of [IrH_2_(POCOP)] is very feasible. The activation free energy of the transition state TS17 is only 6.9 kcal mol^−1^ higher than that of the reacting species ([IrH_2_(POCOP)] + CO_2_). It is noteworthy that the equatorial hydride approaches the C=O bond of CO_2_ without a large change in the configuration around the iridium center in the optimized structure of the transition state TS17. The generated intermediate IM17 is the starting point for the silylation of formate. First, the η^2^-HCOO moiety rotates around the iridium atom to the η^1^-HCOO moiety to accommodate a free silane (TS18 → IM18). Subsequently, a silane molecule coordinates to the iridium atom, which then undergoes a metathesis process of the four-membered ring transition state (TS20), generating silylformate. This metathesis process is feasible, with an activation free energy of 16.7 kcal mol^−1^ (relative to IM19). This barrier would be easily surmountable at the temperatures typically used for the silylation reaction. The potential energy surface in Fig. 7 reveals that the reduction of CO_2_ by the iridium dihydride with silanes is energetically much more preferable. More importantly, this rate-determining barrier is much lower than that calculated for the reduction of CO_2_ to silylformate substrate with the cationic iridium complex (Fig. 4, 29.5 kcal mol^−1^). Thus, we propose that the generation of iridium dihydride [IrH_2_(POCOP)] plays an important role in the reduction of carbon dioxide with silanes catalyzed by the cationic Ir-pincer complex.

**Scheme 4:**
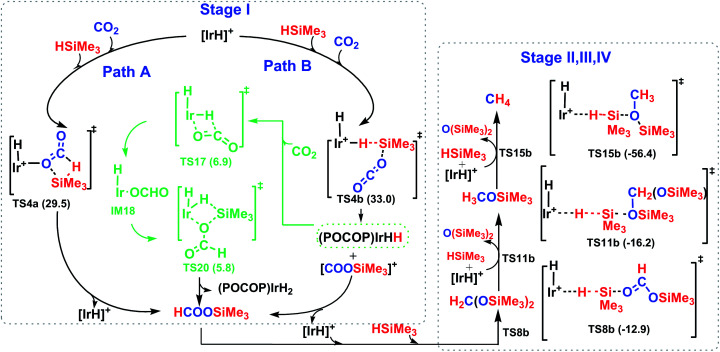
The overall four stages of the reaction mechanism for the catalytic hydrosilylation of CO_2_ to methane catalyzed by the cationic iridium complex.

The overall catalytic cycle for the cationic Ir-pincer complex catalyzing the reduction of CO_2_ with silanes to methane is summarized in Scheme 4. According to our calculations, the whole transformation of CO_2_ with silanes to methane by the cationic Ir-pincer can be divided into four reducing steps with silane hydrogen atoms subsequently being transferred to a carbon dioxide molecule: CO_2_ → silylformate (HCOOSiMe_3_) → bis(silyl)acetal (H_2_C(OSiMe_3_)_2_) → methoxysilane (H_3_COSiMe_3_) → methane (CH_4_). The results obtained in this study are consistent with the catalytic cycle proposed by Brookhart. The first step of reducing CO_2_ to silylformate is the rate-determining step of the overall catalytic cycle. Our DFT results identified two competing pathways: a dissociation pathway featuring the silane Si–H bond directly dissociating onto the C=O bond of an Ir–CO_2_ moiety (passing TS4a) and an ionic S_N_2 outer-sphere pathway of CO_2_ nucleophilically attacking the η^1^-silane iridium complex (passing TS4b and TS7b). Moreover, on the basis of the calculated energy profiles, the generation of the iridium dihydride complex was found to effectively promote CO_2_ hydrosilylation. Our results reveal that the rate-determining step for the CO_2_ activation to silylformate, catalyzed by iridium dihydride, possesses a barrier of 16.7 kcal mol^−1^. The subsequent stages of reducing silylformate to bis(silyl)acetal, methoxysilane and finally to methane are all feasible. The rate-determining activation free energy for stage II of reducing silylformate to bis(silyl)acetal is 12.2 kcal mol^−1^ (TS8b relative to the iridium–silylformate complex). The rate-determining activation free energy for stage III of reducing bis(silyl)acetal to methoxysilane is 16.4 kcal mol^−1^ (TS11b relative to the η^1^-silane iridium complex and bis(silyl)acetal). Furthermore, the rate-determining activation free energy for stage IV of reducing methoxysilane to methane is 22.9 kcal mol^−1^ (TS15b relative to the η^1^-silane iridium complex and methoxysilane).

## Conclusions

The mechanisms behind the cationic Ir-pincer complex catalyzing the hydrosilylation of carbon dioxide to methane product were elucidated using DFT calculations. The calculated results indicate that the conversion of carbon dioxide to methane includes four stages. The first stage of reducing CO_2_ to silylformate is the rate-determining step in the overall carbon dioxide conversion process. The present computational study suggests two possible pathways: the dissociation pathway, in which a free silane dissociates into the weak C=O bond of the Ir–CO_2_ moiety, and the ionic S_N_2 outer-sphere pathway, in which CO_2_ nucleophilically attacks the η^1^-silane iridium complex to cleave the Si–H bond, followed by the hydride transfer process.

Reducing carbon dioxide to silylformate *via* dissociation of the silane Si–H bond to the C=O bond of Ir–CO_2_ has a free energy barrier of around 30 kcal mol^−1^ in solvent. To the best of our knowledge, the pre-coordination of CO_2_ to the metal center being the rate-determining step in a dissociation pathway has not been reported before for M–H complexes. In the second stage, silylformate is reduced to bis(silyl)acetal substrate. The rate-limiting step is calculated to have a free energy barrier of around 12.2 kcal mol^−1^ in solvent. In the third stage, bis(silyl)acetal is reduced to methoxysilane, and the rate-limiting step is calculated to have a free energy barrier of around 16.4 kcal mol^−1^ in solvent. In the fourth stage, methoxysilane is reduced to methane, and the rate-limiting step is calculated to have a free energy barrier of around 22.9 kcal mol^−1^ in solvent. Based on the DFT calculations, the three subsequent reduction steps favor the ionic S_N_2 outer-sphere pathways. Furthermore, our calculations indicate that formaldehyde is unlikely to be an intermediate in the CO_2_ conversion catalyzed by the cationic Ir-pincer complex. The possible ways to generate formaldehyde are all associated with high free energy barriers: 38.7 kcal mol^−1^ (TS6a relative to IM5), 29.2 kcal mol^−1^ (TS8bi relative to IM5), and 34.1 kcal mol^−1^ (TS7c relative to IM5). Moreover, our results show that the *in situ* generation of iridium dihydride can greatly promote the silylation of CO_2_. The computed potential energy barrier for iridium dihydride catalyzing the silylation of CO_2_ is quite low, with an activation free energy of 16.7 kcal mol^−1^.

## Conflicts of interest

There are no conflicts to declare.

## Supplementary Material

RA-008-C7RA13486J-s001

## References

[cit1] (a) Aresta M. , Carbon Dioxide: Utilization Options to Reduce its Accumulation in the Atmosphere, in Carbon Dioxide as Chemical Feedstock, Wiley-VCH, Weinheim, 2010, ch. 1

[cit2] Aresta M., Dibenedetto A. (2007). Dalton Trans..

[cit3] Motokura K., Kashiwame D., Miyaji A., Baba T. (2012). Org. Lett..

[cit4] Sébastien B., Laure V., Sylviane S. (2014). J. Am. Chem. Soc..

[cit5] Porosoff M. D., Yan B. H., Chen J. G. G. (2016). Energy Environ. Sci..

[cit6] Jansen A., Pitter S. (2004). J. Mol. Catal. A: Chem..

[cit7] Sakakura T., Choi J.-C., Yasuda H. (2007). Chem. Rev..

[cit8] Benson E. E., Kubiak C. P., Sathrum A. J., Smieja J. M. (2009). Chem. Soc. Rev..

[cit9] Leitner W. (1995). Angew. Chem., Int. Ed..

[cit10] Graf E., Leitner W. (1992). J. Chem. Soc., Chem. Commun..

[cit11] Jessop P. G., Hsiao Y., Ikariya T., Noyori R. (1996). J. Am. Chem. Soc..

[cit12] Metsanen T. T., Oestreich M. (2015). Organometallics.

[cit13] Scheuermann M. L., Semproni S. P., Pappas I., Chirik P. J. (2014). Inorg. Chem..

[cit14] Gonzalez-Sebastián L., Flores-Alamo M., García J. J. (2013). Organometallics.

[cit15] Süss-Fink G., Reiner J. (1981). J. Organomet. Chem..

[cit16] Séverine M., Dyson P. J., Laurenczy G. (2014). Nat. Commun..

[cit17] Ziebart C., Federsel C., Anbarasan P., Jackstell R., Baumann W., Spannenberg A., Beller M. (2012). J. Am. Chem. Soc..

[cit18] Tanaka R., Yamashita M., Nozaki K. (2009). J. Am. Chem. Soc..

[cit19] Filonenko G. A., Hensen E. J. M., Pidko E. A. (2014). Catal. Sci. Technol..

[cit20] Rohmann K., Kothe J., Haenel M. W., Englert U., Hçlscher M., Leitner W. (2016). Angew. Chem., Int. Ed..

[cit21] Kang P., Cheng C., Chen Z., Schauer C. K., Meyer T. J., Brookhart M. (2012). J. Am. Chem. Soc..

[cit22] Ahn S. T., Bielinski E. A., Lane E. M., Chen Y., Bernskoetter W. H., Hazari N., Palmore G. T. R. (2015). Chem. Commun..

[cit23] Ezhova N. N., Kolesnichenko N. V., Bulygin A. V., Slivinskii E. V., Han S. (2002). Russ. Chem. Bull..

[cit24] Wang W. H., Himeda Y., Muckerman J. T., Manbeck G. F., Fujita E. (2015). Chem. Rev..

[cit25] Cokoja M., Bruckmeier C., Rieger B., Herrmann W. A., Kühn F. E. (2011). Angew. Chem., Int. Ed..

[cit26] Fernandez-Alvarez F. J., Aitani A. M., Oro L. A. (2014). Catal. Sci. Technol..

[cit27] Jiang Y., Blacque O., Fox T., Berke H. (2013). J. Am. Chem. Soc..

[cit28] Berkefeld A., Piers W. E., Parvez M., Castro L., Maron L., Eisenstein O. (2013). Chem. Sci..

[cit29] Deglmann P., Ember E., Hofmann P., Pitter S., Walter O. (2007). Chem.–Eur. J..

[cit30] Motokura K., Kashiwame D., Takahashi N., Miyaji A., Baba T. (2013). Chem.–Eur. J..

[cit31] Jansen A., Görls H., Pitter S. (2000). Organometallics.

[cit32] Ashley A. E., Thompson A. L., O'Hare D. (2009). Angew. Chem., Int. Ed..

[cit33] Holbrey J. D., Reichert W. M., Tkatchenko I., Bouajila E., Walter O., Tommasi I., Rogers R. D. (2003). Chem. Commun..

[cit34] Park S., Bezier D., Brookhart M. (2012). J. Am. Chem. Soc..

[cit35] Kumar N., Camaioni D. M., Dupuis M., Raugei S., Appel A. M. (2014). Dalton Trans..

[cit36] Jessop P. G., Joób F., Taic C. (2004). Coord. Chem. Rev..

[cit37] Jessop P. G., Ikariya T., Noyori R. (1994). Nature.

[cit38] Klankermayer J., Leitner W. (2016). Philos. Trans. A Math. Phys. Eng. Sci..

[cit39] Iglesias M., Oro L. A. (2014). ChemCatChem.

[cit40] Khandelwal M., Wehmschulte R. J. (2012). Angew. Chem., Int. Ed..

[cit41] Becke A. D. (1993). J. Chem. Phys..

[cit42] Yan Z., Truhlar D. G. (2008). J. Phys. Chem. A.

[cit43] Frisch M. J. , et al., Gaussian 09, Revision A.02, Gaussian, Inc., Wallingford, CT, 2009

[cit44] Hay P. J., Wadt W. R. (1985). J. Chem. Phys..

[cit45] Ehlers A. W., Böhme M., Dapprich S., Gobbi A., Hoöllwarth A., Jonas V., Koöhler K. F., Stegmenn R., Frenking G. (1993). Chem. Phys. Lett..

[cit46] Marenich A. V., Cramer C. J., Truhlar D. G. (2009). J. Phys. Chem. B.

[cit47] Grimme S. (2006). J. Comput. Chem..

[cit48] Legault C. Y. , CYLview, 1.0b, Université de Sherbrooke, 2009, http://www.cylview.org

[cit49] Yang J., White P. S., Schauer C. K., Brookhart M. (2008). Angew. Chem., Int. Ed..

[cit50] Park S., Brookhart M. (2010). Organometallics.

[cit51] Gutsulyak D. V., Vyboishchikov S. F., Nikonov G. I. (2010). J. Am. Chem. Soc..

[cit52] Marcos R., Xue L., Sánchez-de-Armas R., Ahlquist Mårten S. G. (2016). ACS Catal..

[cit53] Martin R. L., Hay P. J., Pratt L. R. (1998). J. Phys. Chem. A.

[cit54] Osadchuk I., Tamm T., Ahlquist M. S. G. (2016). ACS Catal..

