# Supplementary material for: Insight into catalytic reduction of CO2 to methane with silanes using Brookhart's cationic Ir(iii) pincer complex
Source: RSC Adv. 2018 Mar 2;8(17):9232–42. doi: 10.1039/c7ra13486j (PMC9078678; doi:10.1039/c7ra13486j)
Supplement: RA-008-C7RA13486J-s001 [file RA-008-C7RA13486J-s001.pdf]

Electronic Supplementary Material (ESI) for RSC Advances.
This journal is © The Royal Society of Chemistry 2018

Supporting Information

Insight Into Catalytic Reduction of CO_2 to Methane with Silanes Using Brookhart's Cationic Ir(III) Pincer Complex

Shaoqin Fang, Hongcai Chen, Haiyan Wei*

Complete reference of 43

Figure S1. The optimized geometries of $\eta^1{}_O$- encountered complex **Ir-CO₂**, the η^1-silane iridium complex **IM2** (The solvent-phase Gibbs energy are given in the bracket), the silylformate iridium intermediate **IM5**, and the intermediate **IM6b**, **TS7b** and the silylformate iridium intermediate **IM7**.
Figure S2. The Optimized geometries of **TS6a**, and **TS6ai** (involves dissociation of a second silane Si–H bond to the C=O bond of silylformate in the silylformate intermediate **IM5**, it should be less favorable than **TS6a**, owe to the scenario of more crowding). **IM10-II** (the intermediate from the transition state **TS6ai**). Bond distances are shown in Å. (The solvent-phase Gibbs energy are given in the bracket).
Figure S3. The schematic represents of stage I of reducing CO_2 to silylformate ($HCOOSiMe_3$) via pathway C (insertion of iridium hydride to the CO_2) and optimized geometries of key stationary points and transition states (**IM3c, IM5c**). The bond distances are shown in Å. The optimized geometries of **IM3c, IM5c** in stage I of reducing CO_2 to silylformate ($HCOOSiMe_3$) via pathway C. Bond distances are shown in Å. (The solvent-phase Gibbs energy are given in the bracket).
Figure S4. The optimized geometries of **IM6c**, **TS7c**, **IM7c**, **IM8c** and **TS9c** in stage II of reducing silylformate to bis(silyl)acetal via pathway C. The bond distances are shown in Å. (The solvent-phase Gibbs energy are given in the bracket).
Figure S5. The optimized geometries of **IM8** (Ir-bound formaldehyde adduct) in stage II of hydrosilylation of silylformate to formaldehyde via pathway A. Bond distances are shown in Å. (The solvent-phase Gibbs energy are given in the bracket).
Figure S6. The optimized geometries of **IM8bi**, **IM9bi** and **IM10bi** in stage II of hydrosilylation of silylformate to formaldehyde via pathway A. Bond distances are shown in Å. (The solvent-phase Gibbs energy are given in the bracket).
Figure S7. Optimized geometries of **IM11b**, **IM12b** and **IM13b** in stage III of reducing bis(silyl)acetal ($H_2C(OSiMe_3)_2$) to methoxysilane ($H_3COSiMe_3$). Bond distances are shown in Å. (The solvent-phase Gibbs energy are given in the bracket).
Figure S8. The Optimized geometries of **TS11bi** in stage III of reducing formaldehyde to methoxysilane. Bond distances are shown in Å. (The solvent-phase Gibbs energy are given in the bracket).
Figure S9. Optimized geometries of **IM14b**, **IM15** in stage IV of reducing methoxysilane to methane (CH_4) under the catalysis of the cation iridium complex. Bond distances are shown in Å. (The solvent-phase Gibbs energy are given in the bracket).
Figure S10. The Optimized geometries of **TS8b-syn**, **TS11b-syn**, **TS14b-syn** (corresponding to the silylformate, bis(silyl)acetal, methoxysilane substrates attack the η^1-silane iridium complex from frontside) via pathway B. Bond distances are shown in Å. (The solvent-phase Gibbs energy are given in the bracket).
Figure S11. The Optimized geometries of the iridium dihydride (POCOP)IrH_2, and the optimized structures of the intermediates and transition states **TS17** , **IM17**, **TS18** , **IM18, IM19, TS20** for reducing CO_2 to silylformate ($HCOOSiMe_3$) with the iridium dihydride complex [IrH_2(POCOP)]. The bond distances are shown in Å. (The solvent-phase Gibbs energy are given in the bracket).
Table S1 Summarize the results from calculations on the reaction of [**Ir**] with CO_2 and a silane of Me_3SiH, Hydrosilylation of CO_2 to Silylformate ($HCOOSiMe_3$). The gas-phase Gibbs free energies (△G) are in kcal/mol .
Table S2 Comparison of the key structures with X-ray single crystal structure of complex with a triethylsilane end-on coordinated to the iridium center as reported by Brookhart and an η^1-silane iridium complex **IM2**.
Table S3 Comparison of the energy barriers of the rate-determining steps of stage I calculated at B3LYP, B3LYP-D and M06 level in the solvent. The solvent-phase Gibbs free energies (△G) are in kcal/mol.
Table S4 Cartesian coordinates for all optimized structures in XYZ format.

Complete reference of 43

Frisch, M. J. et al. *Gaussian 09*; Gaussian, Inc. Frisch, M. J.; Trucks, G. W.; Schlegel, H. B.; Scuseria, G. E.; Robb, M. A.; Cheeseman, J. R.; Montgomery, Jr., J. A.; Vreven, T.; Kudin, K. N.; Burant, J. C.; Millam, J. M.; Iyengar, S. S.; Tomasi, J.; Barone, V.; Mennucci, B.; Cossi, M.; Scalmani, G.; Rega, N.; Petersson, G. A.; Nakatsuji, H.; Hada, M.; Ehara, M.; Toyota, K.; Fukuda, R.; Hasegawa, J.; Ishida, M.; Nakajima, T.; Honda, Y.; Kitao, O.; Nakai, H.; Klene, M.; Li, X.; Knox, J. E.; Hratchian, H. P.; Cross, J. B.; Bakken, V.; Adamo, C.; Jaramillo, J.; Gomperts, R.; Stratmann, R. E.; Yazyev, O.; Austin, A. J.; Cammi, R.; Pomelli, C.; Ochterski, J. W.; Ayala, P. Y.; Morokuma, K.; Voth, G. A.; Salvador, P.; Dannenberg, J. J.; Zakrzewski, V. G.; Dapprich, S.; Daniels, A. D.; Strain, M. C.; Farkas, O.; Malick, D. K.; Rabuck, A. D.; Raghavachari, K.; Foresman, J. B.; Ortiz, J. V.; Cui, Q.; Baboul, A. G.; Clifford, S.; Cioslowski, J.; Stefanov, B. B.; Liu, G.; Liashenko, A.; Piskorz, P.; Komaromi, I.; Martin, R. L.; Fox, D. J.; Keith, T.; Al-Laham, M. A.; Peng, C. Y.; Nanayakkara, A.; Challacombe, M.; Gill, P. M. W.; Johnson, B.; Chen, W.; Wong, M. W.; Gonzalez, C.; and Pople, J. A.; Gaussian, Inc., Pittsburgh PA,, 2009.

Figure S1. The optimized geometries of η[1]$_O$- encountered complex **Ir-CO$_2$** and the η[1]-silane iridium complex **IM2** (The solvent-phase Gibbs energy are given in the bracket), the silylformate iridium intermediate **IM5**, and the intermediatr **IM6b**, **TS7b** and the silylformate iridium intermediate **IM7**.

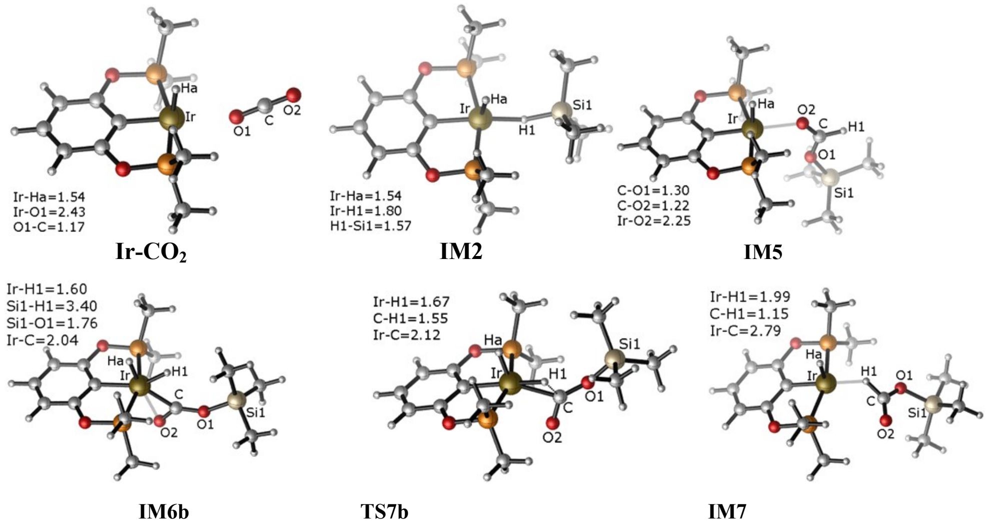

Figure S2. The Optimized geometries of **TS6a**, and **TS6ai** (involves dissociation of a second silane Si–H bond to the C=O bond of silylformate in the silylformate intermediate **IM5**, it should be less favorable than **TS6a**, owe to the scenario of more crowding). **IM10-II** (the intermediate from the transition state **TS6ai**). Bond distances are shown in Å. (The solvent-phase Gibbs energy are given in the bracket).

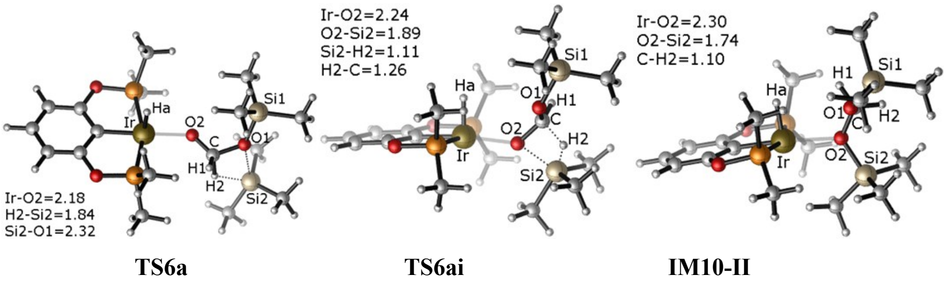

Figure S3. The schematic represents of stage I of reducing CO_2 to silylformate ($HCOOSiMe_3$) via pathway C (insertion of iridium hydride to the CO_2) and optimized geometries of key stationary points and transition states (**IM3c, IM5c)**. The bond distances are shown in Å. The optimized geometries of **IM3c, IM5c** in stage I of reducing CO_2 to silylformate ($HCOOSiMe_3$) via pathway C. Bond distances are shown in Å. (The solvent-phase Gibbs energy are given in the bracket).

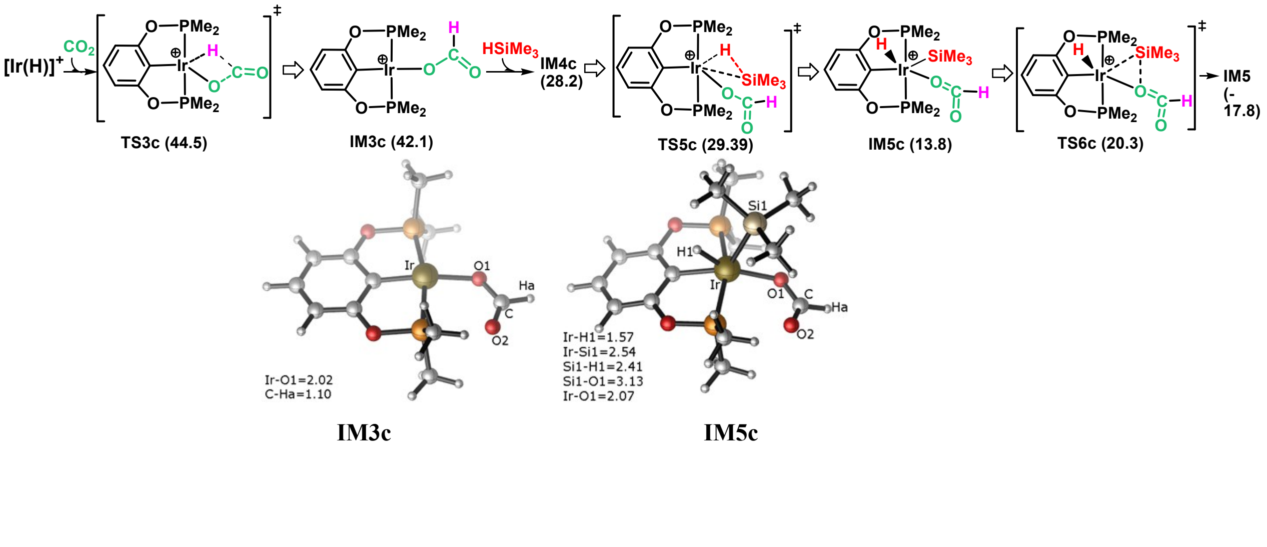

Figure S4. The Optimized Geometries of **IM6c**, **TS7c**, **IM7c**, **IM8c**, and **TS9c** in stage II of reducing silylformate to bis(silyl)acetal via pathway C. The bond distances are shown in Å. (The solvent-phase Gibbs energy are given in the bracket).

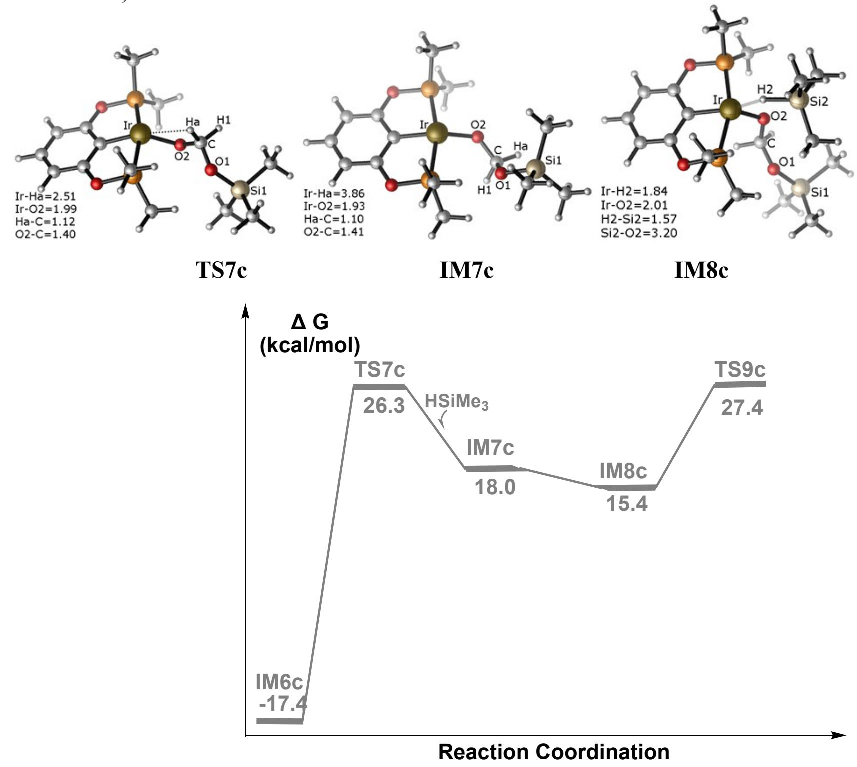

Figure S5. The optimized geometries of **IM8** (Ir-bound formaldehyde adduct) in stage II of hydrosilylation of silylformate to formaldehyde via pathway A. Bond distances are shown in Å. (The solvent-phase Gibbs energy are given in the bracket).

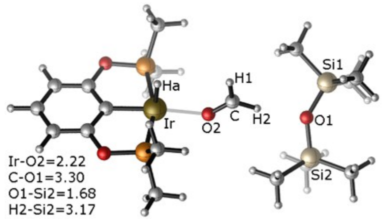

IM8

Figure S6. The optimized geometries of **IM8bi**, **IM9bi** and **IM10bi** in stage II of hydrosilylation of silylformate to formaldehyde via pathway A. Bond distances are shown in Å. (The solvent-phase Gibbs energy are given in the bracket).

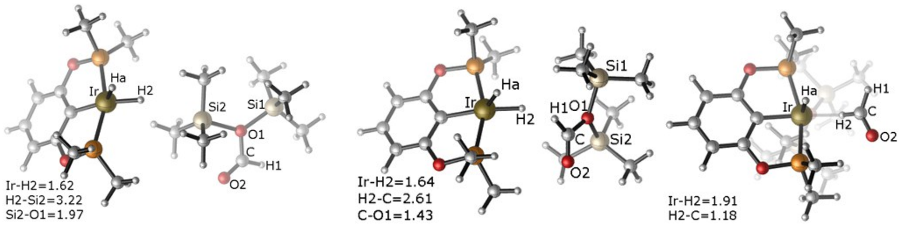

IM8bi **IM9bi** **IM10bi**

Figure S7. Optimized geometries of **IM11b**, **IM12b** and **IM13b** in stage III of reducing bis(silyl)acetal ($H_2C(OSiMe_3)_2$) to methoxysilane ($H_3COSiMe_3$). Bond distances are shown in Å. (The solvent-phase Gibbs energy are given in the bracket).

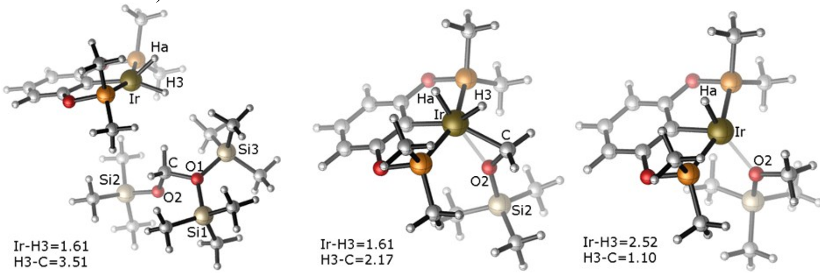

IM11b **IM12b** **IM13b**

Figure S8. The Optimized geometries of **TS11bi** in stage III of reducing formaldehyde to methoxysilane. Bond distances are shown in Å. (The solvent-phase Gibbs energy are given in the bracket).

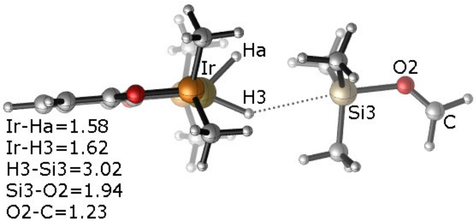

TS11bi

Figure S9. Optimized geometries of **IM14b**, **IM15** in stage IV of reducing methoxysilane to methane (CH_4) under the catalysis of the cation iridium complex. Bond distances are shown in Å. (The solvent-phase Gibbs energy are given in the bracket).

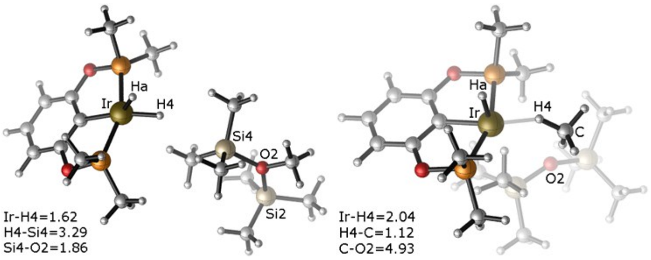

IM14b **IM15**

Figure S10. The Optimized geometries of **TS8b-syn**, **TS11b-syn**, **TS14b-syn** (corresponding to the silylformate，bis(silyl)acetal，methoxysilane substrates attack the η^1-silane iridium complex from frontside) via pathway B. Bond distances are shown in Å. (The solvent-phase Gibbs energy are given in the bracket).

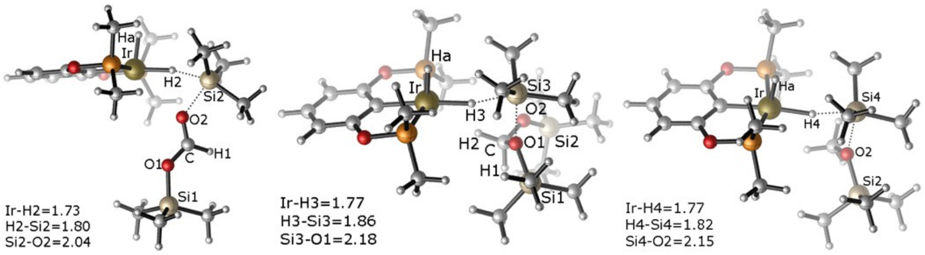

TS8b-syn (9.6) **TS11b-syn (-3.2)** **TS14b-syn(-40.1)**

Figure S11. The Optimized geometries of the iridium dihydride (POCOP)IrH_2, and the optimized structures of the intermediates and transition states **TS17** , **IM17**, **TS18** , **IM18, IM19, TS20** for reducing CO_2 to silylformate ($HCOOSiMe_3$) with the iridium dihydride complex [IrH_2(POCOP)]. The bond distances are shown in Å. (The solvent-phase Gibbs energy are given in the bracket).

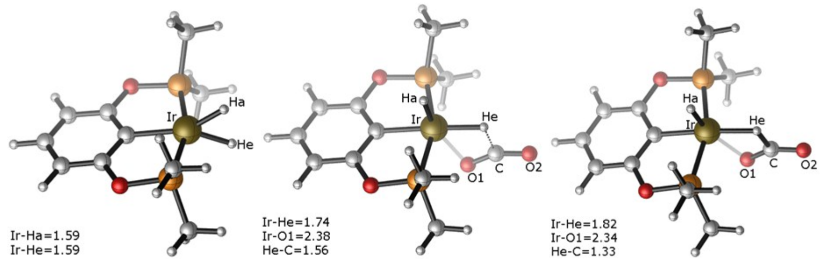

(POCOP)IrH$_2$ **TS17** **IM17**

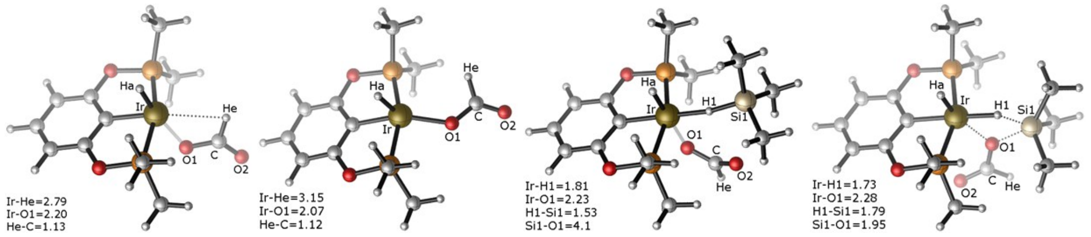

TS18 **IM18** **IM19** **TS20**

Table S1 Summarizes the results from calculations on the reaction of [**Ir**] with CO_2 and a silane of Me_3SiH, Hydrosilylation of CO_2 to Silylformate ($HCOOSiMe_3$). The gas-phase Gibbs free energies (ΔG) are in kcal/mol.

pathwayA	**Ir-CO$_2$**	**TS4a**	**IM4**	**TS5a**	**IM5**	
B3LYP/6-311Gdp Gas-phase	-3.4	22.0	-19.4	-15.5	-26.7	
pathwayB	**IM2**	**TS4b**	**IM6b**	**TS7b**	**IM7**	
B3LYP/6-311Gdp Gas-phase	-19.0	23.6	-8.0	-4.6	-12.1	
pathwayC	**TS3c**	**IM3c**	**IM4c**	**TS5c**	**IM5c**	**TS6c**
B3LYP/6-311Gdp Gas-phase	44.4	25.5	14.2	16.0	0.3	9.8

Table S2 Comparison of the key structures with X-ray single crystal structure of complex with a triethylsilane end-on coordinated to the iridium center as reported by Brookhart and an η^1-silane iridium complex **IM2**.

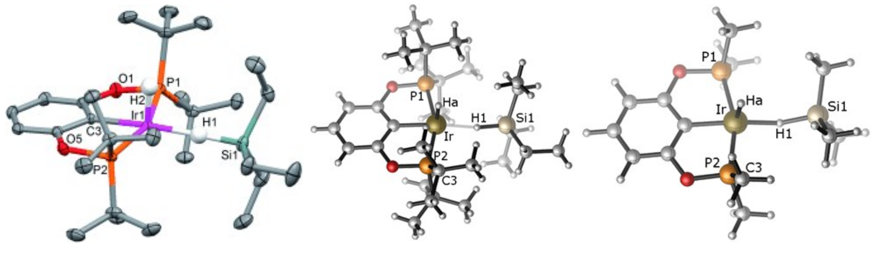

Ir-HSiEt$_3$ **IM2**

Distances/angles	Ir-Ha	Ir-H1	H1-Si1	Ir-Si1	Ir-P1	Ir-P2	Ir-C3	Ir-H1-Si1	P2-Ir-P1

**Ir-HSiEt$_3$**	1.43	1.94	1.48	3.35	2.35	2.31	2.02	157	158
**M2**	1.54	1.80	1.57	3.27	2.34	2.34	3.63	152	160

Table S3 Comparison of the energy barriers of the rate-determining steps of stage I calculated at B3LYP, B3LYP-D and M06 level in the solvent. The solvent-phase Gibbs free energies (ΔG) are in kcal/mol. (The correction according to Martin is not included here)

	**TS3a**	**TS4a**	**TS3b**	**TS4b**	**TS5b**
**B3LYP**	48.1	49.8	52.5	55.4	57.7
**B3LYP-D**	39.2	38.1	42.5	41.6	42.4
**M06**	42.6	44.2	45.2	47.1	47.7

Table S4 Cartesian coordinates for all optimized structures in XYZ format.

1Ir [(POCOP)Ir(H)(acetone)]$^+$

1Ir

C	-0.038714	3.883762	-0.017258
C	1.184740	3.213649	-0.009796
C	1.260852	1.821033	0.018055
C	0.082414	1.090815	0.035683
C	-1.170698	1.729910	0.004262
C	-1.206399	3.136477	0.000662
O	0.142418	-0.288632	0.093554
P	-1.311477	-1.102462	0.047755
C	-1.106691	-2.205005	-1.386312
Ir	-2.834438	0.680980	-0.038726
P	-3.786458	2.825937	-0.019498
C	-4.670182	3.451125	-1.483145
O	-2.425397	3.787118	0.023778
C	-4.740216	3.400696	1.415610
C	-1.227166	-2.175372	1.511072
H	-1.041493	-1.615434	-2.301722
H	-0.197710	-2.800163	-1.275603
H	-1.231373	-1.563421	2.413336
H	-0.317246	-2.778319	1.483592
H	-2.097854	-2.835467	1.527068
H	-1.968998	-2.872571	-1.457162
H	-4.816090	4.530335	-1.401100
H	-4.096205	3.233142	-2.384729
H	-5.643484	2.959737	-1.557011
H	-4.890610	4.480727	1.359318
H	-4.204092	3.155010	2.332557
H	-5.711742	2.900656	1.428866
H	-3.100376	0.539347	1.474970
H	-0.094030	4.964399	-0.029975
H	2.101130	3.790791	-0.021694
H	2.211703	1.304636	0.032680

Ir-CO2

C	-1.107650	3.109461	-0.021045
C	0.086211	3.851039	-0.072528
C	1.295639	3.133397	-0.060608
C	1.325642	1.747508	-0.039280
C	0.115068	1.055273	-0.010503
C	-1.109336	1.723257	0.000809
Ir	0.065082	5.828148	-0.157421
P	-2.245598	5.441990	-0.069413
C	-3.227881	5.962798	1.369721
O	2.493135	3.830988	-0.060163
P	2.384814	5.488102	-0.145739
C	3.403757	6.028709	1.260223
O	-2.318146	3.783092	0.019020
C	3.366624	5.873404	-1.631902
C	-3.283533	5.807371	-1.522080
H	-2.808111	5.417943	-2.423363
H	-4.266630	5.345839	-1.407392
H	-2.707440	5.685268	2.286622
H	-4.207134	5.480132	1.348411
H	-3.361485	7.047035	1.350984
H	-3.401931	6.888646	-1.625188
H	4.361958	5.431549	-1.549925
H	2.869445	5.474154	-2.517005
H	3.460010	6.956767	-1.738686
H	4.390973	5.565162	1.206417
H	2.919454	5.741470	2.193784
H	3.515543	7.115325	1.237305
H	0.088028	6.048306	1.368209
H	2.275460	1.229184	-0.038928
H	0.126157	-0.027588	0.007823
H	-2.048120	1.186125	0.032251
O	0.036353	8.241009	-0.441760
C	0.032391	9.324709	-0.001484
O	0.028261	10.395181	0.419127

M2

C	-1.243020	3.077732	0.140495
C	-0.089129	3.846009	0.178087
C	1.143317	3.194961	0.121225
C	1.234083	1.805880	0.030507
C	0.059615	1.069305	-0.004853
C	-1.207949	1.676307	0.033187
O	0.137468	-0.311690	-0.073529
P	-1.310953	-1.129687	-0.163038
C	-1.163257	-2.309565	1.214833
Ir	-2.893972	0.592759	-0.102713
P	-3.815707	2.731646	0.124668
C	-4.726823	3.196931	1.629988
O	-2.471929	3.711741	0.217413
C	-1.083438	-2.127271	-1.672712
C	-4.739851	3.495595	-1.249484
H	-1.054093	-1.474079	-2.545817
H	-0.147996	-2.687461	-1.612803
H	-1.191641	-1.767344	2.160304
H	-0.218085	-2.850813	1.137484
H	-1.990857	-3.020605	1.186637

H	-1.916589	-2.825169	-1.781709
H	-4.859143	4.566092	-1.069896
H	-4.199082	3.347779	-2.185193
H	-5.724923	3.031185	-1.333900
H	-4.846440	4.281542	1.669328
H	-4.170343	2.863656	2.506363
H	-5.710160	2.723376	1.632076
H	-3.107741	0.340482	1.402863
H	-0.157829	4.923148	0.254387
H	2.052763	3.782725	0.150461
H	2.190849	1.301979	-0.007519
H	-4.369500	-0.332019	-0.542930
Si	-5.639475	-1.175889	-0.181171
C	-5.074679	-2.783138	0.594418
C	-6.725118	-0.172898	0.967529
C	-6.391995	-1.407123	-1.880948
H	-7.631983	-0.744150	1.191860
H	-6.229890	0.042540	1.916854
H	-7.042798	0.768924	0.513493
H	-5.948204	-3.414731	0.786698
H	-4.409364	-3.346204	-0.064732
H	-4.571618	-2.620753	1.550222
H	-7.311871	-1.995596	-1.802049
H	-6.650378	-0.451273	-2.343252
H	-5.716636	-1.938653	-2.555896

TS3a

C	-0.325201	1.249977	1.072920
C	-0.685661	2.417440	0.415766
C	0.184959	3.512979	0.297664
C	1.444140	3.395380	0.908296
C	1.840252	2.243868	1.573960
C	0.944493	1.177015	1.646632
O	-1.958399	2.511734	-0.136849
P	-2.310749	3.933626	-0.930031
C	-2.825331	3.340057	-2.576558
Ir	-0.344791	5.163402	-0.691411
P	1.788090	5.805578	-0.006220
C	3.124295	6.017540	-1.229611
O	2.316342	4.472991	0.851263
C	-3.852597	4.467164	-0.121962
C	2.018475	7.156963	1.189647
O	-0.977972	6.897118	-1.984544
C	-0.376020	7.963839	-2.148770
O	0.685501	8.443397	-1.757428
H	-1.972070	2.888766	-3.084821
H	-3.621003	2.597845	-2.484606
H	-3.643472	4.733226	0.914419
H	-4.593070	3.665192	-0.148139
H	-4.246646	5.344702	-0.639600
H	-3.180804	4.186162	-3.169135
H	4.093039	6.063971	-0.727854
H	3.121841	5.175226	-1.922982
H	2.946336	6.940808	-1.782600
H	3.043575	7.159983	1.565375
H	1.328235	7.022788	2.022898
H	1.801020	8.101244	0.688631
H	-0.928493	5.838059	0.562378
H	2.821839	2.191089	2.026561
H	1.240065	0.272420	2.164190
H	-1.023865	0.426097	1.137079
H	-1.018710	8.656387	-2.868093
Si	-0.247174	10.385759	-3.197910
C	-1.781542	10.667971	-4.220297
C	-0.215362	11.322646	-1.602618
C	1.293897	10.137536	-4.191372
H	-1.716406	11.680859	-4.640097
H	-1.862517	9.971114	-5.057120
H	-2.696364	10.618579	-3.626008
H	1.429219	11.015076	-4.835565
H	2.169129	10.023583	-3.553417
H	1.215417	9.266370	-4.845892
H	-0.324609	12.390084	-1.830324
H	-1.050311	11.043417	-0.955786
H	0.717254	11.164382	-1.063041

TS4a

Ir	0.184303	-0.053959	0.176912
P	0.304221	0.227450	2.501176
O	1.928039	0.540323	2.744438
C	2.725344	0.579642	1.614757
C	4.083948	0.824771	1.762387
C	4.879205	0.881833	0.618730
C	4.336764	0.702484	-0.654081
C	2.975876	0.459277	-0.760416
C	2.130720	0.373712	0.358086
O	2.423597	0.296585	-2.026654
P	0.787440	0.007082	-2.077826
C	0.190661	1.368971	-3.134355
C	0.729146	-1.467219	-3.156687
H	-0.285343	1.407143	0.063229
C	0.040661	-1.160728	3.654806
C	-0.477909	1.668590	3.290640
O	-1.955882	-0.761860	-0.068325
Si	-3.962670	-1.812864	-1.215925
C	-3.894814	-0.375285	-2.382995
C	-2.870373	-3.291642	-1.445914
C	-5.690653	-2.241062	-0.658181
C	-2.777161	-0.712488	0.903286
H	-3.830362	-1.128040	0.585401
O	-2.709042	-0.350937	2.045303
H	0.562698	-2.047227	3.291875
H	0.433477	-0.895242	4.638501
H	-0.237431	2.565914	2.719829
H	-0.101680	1.778340	4.309721
H	-1.558058	1.524937	3.305519
H	-1.026912	-1.369018	3.727501
H	1.312413	-1.291944	-4.063097
H	1.145169	-2.325686	-2.627555
H	-0.304958	-1.685565	-3.433263
H	0.744140	1.391110	-4.075245
H	0.333898	2.314088	-2.609692
H	-0.874318	1.243665	-3.342018
H	4.949666	0.753671	-1.544430
H	5.940697	1.072699	0.720005
H	4.501215	0.970974	2.750079
H	-3.341794	-3.960220	-2.176284
H	-1.878085	-3.016672	-1.800310
H	-2.765778	-3.854529	-0.515198
H	-4.539589	-0.594894	-3.242280
H	-4.278727	0.533789	-1.914006
H	-2.882781	-0.184593	-2.735766
H	-6.204904	-2.711280	-1.507164
H	-5.700434	-2.955824	0.167441
H	-6.269015	-1.360673	-0.370130

IM4

C	-1.102477	1.700981	0.060359
C	-1.103167	3.085703	-0.020827
C	0.081808	3.833661	-0.114728
C	1.288835	3.117455	-0.078664

C	1.329452	1.733340	0.001530
C	0.123384	1.036072	0.066087
O	-2.318968	3.756504	-0.001670
P	-2.241100	5.417637	-0.036782
C	-3.483170	5.801372	-1.317397
Ir	0.049795	5.820841	-0.282957
P	2.362283	5.478932	-0.174496
C	3.515696	5.865730	-1.535332
O	2.485335	3.821144	-0.117342
C	-3.023116	5.913163	1.530598
C	3.234505	6.015739	1.331004
O	-0.015371	8.160086	-0.684486
C	-0.133160	8.961257	0.417835
O	-0.224813	8.531218	1.524353
H	-3.135948	5.455867	-2.291851
H	-4.424315	5.300906	-1.079656
H	-2.412851	5.559014	2.361726
H	-4.026398	5.489637	1.606985
H	-3.076959	7.002758	1.583643
H	-3.653347	6.879759	-1.356041
H	4.481325	5.392398	-1.345056
H	3.119267	5.489793	-2.479247
H	3.656529	6.946435	-1.607632
H	4.245705	5.604756	1.352678
H	2.683634	5.673316	2.207236
H	3.282909	7.106877	1.355758
H	0.086015	6.097900	1.226431
H	2.282374	1.220980	0.020108
H	0.139233	-0.045082	0.129645
H	-2.039424	1.163468	0.124766
Si	0.039169	8.810824	-2.344218
C	0.054272	7.281920	-3.414325
C	-1.507283	9.832373	-2.576759
C	1.604868	9.819250	-2.481254
H	0.030714	7.579736	-4.468139
H	0.955482	6.680244	-3.275602
H	-0.820966	6.648743	-3.245783
H	1.700424	10.214245	-3.497688
H	1.611819	10.676816	-1.803144
H	2.497375	9.221442	-2.279539
H	-1.552648	10.195803	-3.608383
H	-2.413428	9.247166	-2.399053
H	-1.538725	10.711770	-1.928196
H	-0.135115	10.033012	0.171363

TS5a

C	-1.100577	1.677854	-0.074996
C	-1.107002	3.063568	-0.007574
C	0.077824	3.814810	-0.041671
C	1.288335	3.108433	-0.097539
C	1.330070	1.723428	-0.166034
C	0.126155	1.019643	-0.157602
O	-2.323017	3.727263	0.098025
P	-2.246586	5.384428	0.224125
C	-3.468976	5.904865	-1.024662
Ir	0.037984	5.802965	0.003670
P	2.354013	5.474870	0.024779
C	3.464215	6.001818	-1.323991
O	2.481677	3.819121	-0.080468
C	-3.032194	5.734767	1.826726
C	3.270560	5.868835	1.547785
O	-0.046827	8.277270	-0.935316
C	-0.286869	8.999958	0.173570
O	-0.419092	8.474189	1.242678
H	-3.107029	5.657208	-2.023243
H	-4.418991	5.394185	-0.853635
H	-2.437938	5.285173	2.622675
H	-4.045574	5.329778	1.855240
H	-3.059040	6.815370	1.983337
H	-3.623903	6.983956	-0.954468
H	4.437774	5.520287	-1.210291
H	3.040855	5.717591	-2.287871
H	3.595181	7.085650	-1.291605
H	4.280478	5.456617	1.501385
H	2.745463	5.447117	2.405042
H	3.323645	6.953171	1.670431
H	0.103014	5.785273	1.536789
H	2.284163	1.215278	-0.217150
H	0.144474	-0.062003	-0.209468
H	-2.035839	1.133786	-0.055737
Si	0.067056	8.889114	-2.593749
C	0.090575	7.315063	-3.595191
C	-1.453807	9.928426	-2.907332
C	1.652056	9.869581	-2.710257
H	0.219605	7.545257	-4.657167
H	0.915535	6.658738	-3.306067
H	-0.844510	6.757163	-3.498175
H	1.781103	10.248036	-3.729175
H	1.653146	10.736468	-2.043569
H	2.528405	9.260050	-2.475623
H	-1.464295	10.256022	-3.951772
H	-2.375042	9.365387	-2.734996
H	-1.485688	10.830968	-2.291054
H	-0.351106	10.086167	0.017048

IM5

C	-0.737067	1.422123	0.013974
C	-0.865590	2.797449	0.141388
C	0.242842	3.649349	0.267463
C	1.507940	3.041979	0.296187
C	1.678752	1.670996	0.171526
C	0.544586	0.871678	0.028153
O	-2.140337	3.354541	0.151814
P	-2.217186	5.007273	0.318062
C	-3.281060	5.475604	-1.088178
Ir	0.029386	5.633044	0.406018
P	2.349064	5.478455	0.609017
C	3.461460	6.164557	-0.664052
O	2.630793	3.846072	0.463092
C	-3.265318	5.227394	1.789896
C	3.140227	5.894634	2.194515
O	-0.106754	8.322776	-1.670786
C	-0.234471	8.665491	-0.420428
O	-0.211524	7.863815	0.504900
H	-2.750107	5.299863	-2.024422
H	-4.199032	4.884219	-1.079454
H	-2.745395	4.836111	2.664667
H	-4.213509	4.700521	1.665662
H	-3.455874	6.292279	1.942804
H	-3.534175	6.535819	-1.015912
H	4.473391	5.776973	-0.528972
H	3.100125	5.885447	-1.654473
H	3.478391	7.253926	-0.585333
H	4.183047	5.571183	2.197285
H	2.605297	5.399816	3.005465
H	3.091953	6.974888	2.350653
H	-0.065037	5.595438	1.939534
H	2.673117	1.244717	0.193963
H	0.661388	-0.200839	-0.068584
H	-1.618659	0.802605	-0.085893
Si	-0.102041	9.290500	-3.152705
C	-1.372697	8.452028	-4.226924

C	-0.562255	11.044618	-2.697722
C	1.638643	9.135507	-3.801790
H	-1.418685	8.938970	-5.205849
H	-1.122358	7.401419	-4.395221
H	-2.372973	8.501513	-3.788859
H	1.732330	9.665138	-4.754897
H	2.370201	9.568321	-3.114195
H	1.909168	8.091545	-3.979746
H	-0.592561	11.645998	-3.612153
H	-1.552198	11.116633	-2.238713
H	0.164802	11.519050	-2.032869
H	-0.370582	9.731527	-0.202052

TS3b

C	3.840364	0.968430	-1.444291
C	3.354636	-0.153599	-0.785059
C	4.023252	-1.388104	-0.815548
C	5.236066	-1.448185	-1.520820
C	5.756860	-0.350284	-2.193767
C	5.041246	0.847813	-2.146507
O	2.178238	-0.055112	-0.063513
P	1.729208	-1.443317	0.779116
C	-0.063559	-1.446703	0.410203
Ir	3.246350	-3.048679	0.119612
P	5.269932	-3.880264	-0.606505
C	5.500113	-5.275786	-1.768253
O	5.936625	-2.641167	-1.533292
C	6.563193	-4.152709	0.653162
C	1.809217	-0.879450	2.513831
C	1.241193	-4.501018	-2.459654
Si	0.629539	-5.932223	-1.493759
C	-0.741624	-5.680904	-0.280909
O	-0.648002	-6.911781	-3.021662
C	-1.122340	-7.001092	-4.093790
O	-1.592007	-7.101719	-5.132547
C	1.731895	-7.395285	-1.252490
H	2.398302	-4.434635	0.390483
H	3.371158	-3.680053	1.548899
H	4.834638	-5.153138	-2.623309
H	6.532150	-5.304924	-2.123591
H	6.597787	-3.287926	1.315734
H	7.537436	-4.291116	0.180385
H	6.311703	-5.035183	1.245478
H	5.267737	-6.213458	-1.258604
H	-0.495861	-0.468170	0.628836
H	-0.219214	-1.674764	-0.644682
H	-0.557903	-2.204142	1.022471
H	1.241010	0.043489	2.644781
H	2.851911	-0.708016	2.781121
H	1.405919	-1.656879	3.166368
H	3.303752	1.907213	-1.402163
H	5.435161	1.714683	-2.664498
H	6.694736	-0.426075	-2.728265
H	1.165490	-8.288909	-0.981566
H	2.336786	-7.606305	-2.136529
H	2.412021	-7.165915	-0.425130
H	-1.246353	-6.618867	-0.039514
H	-0.300562	-5.290498	0.642519
H	-1.477675	-4.956499	-0.635002
H	0.475781	-3.783033	-2.753607
H	1.867256	-4.046915	-1.653577
H	1.885010	-4.754642	-3.301607

TS4b

C	0.238662	1.202861	-0.073945
C	-1.045752	1.767641	-0.035087
C	-1.118199	3.166583	0.052452
C	0.010382	3.974198	0.074235
C	1.261590	3.358357	0.017591
C	1.394509	1.970849	-0.054869
Ir	-2.735039	0.606861	-0.148474
P	-1.090569	-1.027061	-0.153010
C	-0.887293	-2.131351	1.285394
O	-2.369561	3.758868	0.129987
P	-3.675641	2.713414	0.090891
C	-4.538145	3.162310	1.635594
O	0.351023	-0.178598	-0.123190
C	-4.677584	3.497199	-1.224025
C	-0.799689	-2.111750	-1.597239
C	-4.393775	-0.545988	-2.902375
O	-3.532608	-0.002741	-3.429367
O	-5.314775	-1.131148	-2.458581
Si	-6.108576	-1.699043	-0.426264
C	-6.757297	-0.200011	0.450828
C	-7.432028	-2.565947	-1.402887
C	-4.932398	-2.886756	0.375901
H	-0.812012	-1.514474	-2.509903
H	0.170412	-2.604899	-1.508416
H	-0.943780	-1.540905	2.200146
H	0.082073	-2.631377	1.238381
H	-1.680567	-2.881495	1.296591
H	-1.584049	-2.869839	-1.655740
H	-4.802789	4.561872	-1.016451
H	-4.175579	3.381056	-2.185552
H	-5.659911	3.022345	-1.274805
H	-4.655239	4.246184	1.694308
H	-3.951393	2.816831	2.487038
H	-5.522534	2.691100	1.666868
H	-3.153548	0.224168	1.284995
H	-0.087152	5.049952	0.139222
H	2.153486	3.973482	0.033785
H	2.367113	1.497753	-0.089754
H	-4.122807	-0.334173	-0.423588
H	-7.692705	-0.486465	0.947520
H	-6.046391	0.177898	1.182243
H	-6.999893	0.595897	-0.257004
H	-5.533076	-3.652260	0.883092
H	-4.321968	-3.401581	-0.369726
H	-4.280358	-2.390868	1.091583
H	-8.114163	-3.042047	-0.687628
H	-8.021644	-1.877506	-2.011360
H	-7.032422	-3.349754	-2.049061

TS5b

C	-3.359746	0.677456	-1.084488
C	-2.566259	-0.272764	-0.425778
C	-3.101637	-1.558813	-0.264880
C	-4.352026	-1.905669	-0.753437
C	-5.094729	-0.927624	-1.416283
C	-4.614857	0.371517	-1.588041
Ir	-0.716851	0.205396	0.296442
P	-1.354647	2.276097	-0.632184
C	-0.468404	2.874930	-2.103839
O	-2.359115	-2.512795	0.413935
P	-0.854973	-2.053859	0.955008
C	0.239741	-3.260428	0.145325
O	-2.876386	1.968893	-1.228628
C	-0.911144	-2.520201	2.710789
C	-1.632467	3.732750	0.418703
Si	2.415067	1.067953	1.663573
C	3.503872	-0.409498	1.999344

C	1.589172	1.569003	3.289547
C	3.157393	2.598282	0.896666
O	2.254432	0.296756	-0.389836
C	1.118530	0.028842	-0.762976
H	-6.074020	-1.182451	-1.803173
H	-5.198886	1.127721	-2.095548
H	-4.732980	-2.908918	-0.616092
H	-0.126413	-4.272686	0.327611
H	1.253998	-3.165342	0.539397
H	0.257638	-3.066310	-0.927611
H	-1.043055	3.669493	-2.583850
H	0.512443	3.259960	-1.816188
H	-0.332914	2.048945	-2.802828
H	-1.631007	-1.889470	3.232886
H	-1.208548	-3.566281	2.808219
H	0.075171	-2.381444	3.158834
H	-2.140270	4.512181	-0.153002
H	-0.674102	4.113998	0.777870
H	-2.247420	3.453221	1.274453
H	-1.350044	0.630007	1.654078
H	0.560577	0.715955	1.287134
H	1.072937	0.737573	3.772937
H	2.387919	1.907732	3.962527
H	4.094608	-0.206876	2.898988
H	0.882488	2.391732	3.166424
H	2.915812	-1.308901	2.201586
H	3.708901	3.141178	1.671578
H	2.387467	3.277360	0.520216
H	3.833950	2.359315	0.076347
H	4.177184	-0.620267	1.168688
O	0.515531	-0.393800	-1.725992

IM6b

C	-3.394082	0.711236	-1.106642
C	-2.627368	-0.215767	-0.416405
C	-3.067891	-1.524949	-0.190343
C	-4.344114	-1.869730	-0.650734
C	-5.144076	-0.974384	-1.345092
C	-4.650596	0.312557	-1.565537
O	-1.386648	0.166295	0.074604
P	-0.470884	-1.005679	0.820309
C	-0.007257	-0.207178	2.384574
Ir	-1.878896	-2.897211	0.782745
C	-0.478014	-4.284814	0.258744
O	-0.623537	-3.974528	-0.920729
O	-4.824746	-3.146611	-0.396121
P	-3.795980	-4.207958	0.367645
C	-4.819073	-4.835170	1.731645
C	1.035026	-1.054161	-0.198279
C	-3.647997	-5.566111	-0.832413
O	0.326058	-5.179777	0.698133
Si	0.909001	-5.993119	2.143617
C	2.737290	-5.635824	2.115626
C	0.491091	-7.779351	1.817681
C	0.068414	-5.327849	3.675238
H	-5.263080	1.024863	-2.105204
H	-3.021578	1.712522	-1.277298
H	-6.121608	-1.273225	-1.699419
H	-4.640111	-5.875450	-1.166755
H	-3.140490	-6.413587	-0.366349
H	-3.059151	-5.230328	-1.686534
H	1.423264	-0.042891	-0.334341
H	1.790794	-1.670891	0.293104
H	0.800310	-1.492842	-1.168542
H	-5.006837	-4.032245	2.444762
H	-5.770262	-5.206436	1.344901
H	-4.291764	-5.645593	2.239262
H	0.449870	0.764367	2.186290
H	0.702751	-0.840477	2.920616
H	-0.895957	-0.070174	3.001016
H	-2.645790	-2.272484	1.990116
H	-1.012446	-5.485534	3.671515
H	0.470465	-5.869554	4.538469
H	0.890521	-8.411800	2.616531
H	0.263027	-4.266232	3.842769
H	-0.588782	-7.945498	1.777233
H	3.239326	-6.170528	2.927856
H	2.947771	-4.570782	2.244988
H	3.189307	-5.960755	1.175353
H	0.924363	-8.122236	0.874994
H	-1.445291	-3.535489	2.182809

TS7b

C	-3.120412	0.798933	-1.026704
C	-2.428506	-0.278770	-0.494253
C	-3.061422	-1.459254	-0.090379
C	-4.454858	-1.502725	-0.223305
C	-5.189470	-0.448977	-0.746975
C	-4.505824	0.698224	-1.147858
O	-1.048383	-0.185957	-0.359412
P	-0.264261	-1.521736	0.225776
C	0.700988	-0.840777	1.606328
Ir	-2.005768	-3.060016	0.647793
C	-0.714317	-4.688042	0.206443
O	-1.199461	-4.875867	-0.890157
O	-5.135441	-2.640791	0.202123
P	-4.213848	-3.877264	0.796407
C	-4.977037	-4.220762	2.408767
C	0.932738	-1.899821	-1.090998
C	-4.628741	-5.281442	-0.280031
O	0.408929	-5.162289	0.635628
Si	1.055402	-5.864442	2.118445
C	1.413073	-4.466561	3.306254
C	2.583848	-6.730096	1.511490
C	-0.265475	-7.025021	2.751636
H	-5.063432	1.530198	-1.560265
H	-2.587202	1.688167	-1.335914
H	-6.265019	-0.527302	-0.834092
H	-5.711833	-5.414720	-0.317635
H	-4.161189	-6.188550	0.108901
H	-4.245036	-5.094909	-1.283130
H	1.509706	-1.006126	-1.336621
H	1.607537	-2.692495	-0.761241
H	0.398330	-2.242670	-1.977983
H	-4.820095	-3.370704	3.073207
H	-6.048467	-4.389717	2.283091
H	-4.519340	-5.108319	2.850746
H	1.308958	-0.004625	1.254811
H	1.349427	-1.615684	2.018280
H	0.023327	-0.492711	2.386164
H	-2.140681	-2.366933	2.023572
H	-0.548689	-7.761240	1.994658
H	0.114104	-7.576547	3.617500
H	3.104597	-7.209728	2.345946
H	-1.166296	-6.497590	3.077559
H	2.336052	-7.504712	0.781860
H	1.805206	-4.876829	4.242401
H	0.518292	-3.889783	3.553435
H	2.172451	-3.786189	2.911811
H	3.282369	-6.033090	1.041694
H	-1.540492	-4.427105	1.490637

IM7

C	-1.382190	0.861902	-1.926693
C	-0.098009	1.016711	-1.426430
C	0.671229	-0.091011	-1.019782
C	0.117888	-1.375242	-1.184432
C	-1.163578	-1.555644	-1.681578
C	-1.900455	-0.427958	-2.046232
O	0.439937	2.285390	-1.332212
P	1.946546	2.404633	-0.622235
C	2.892637	3.379813	-1.826775
Ir	2.470441	0.151341	-0.230606
O	4.631304	-0.085427	2.480705
Si	5.175634	0.168089	4.157804
C	6.835564	1.014585	4.070262
O	0.875029	-2.483680	-0.851856
P	2.384692	-2.185824	-0.204879
C	3.479267	-3.169246	-1.272328
C	4.445073	0.864011	1.600813
O	4.526036	2.054058	1.736932
C	2.318017	-3.078453	1.380793
C	1.641094	3.514724	0.785775
C	3.861689	1.180678	5.016284
C	5.271685	-1.587393	4.780270
H	-2.901923	-0.558230	-2.438023
H	-1.566446	-2.554359	-1.786500
H	-1.954562	1.732121	-2.219797
H	1.199018	4.451064	0.439506
H	2.590538	3.709601	1.287852
H	0.960674	3.032596	1.489156
H	2.061755	-4.127192	1.217437
H	3.292775	-3.008456	1.868628
H	1.568377	-2.620394	2.027298
H	3.028419	2.803101	-2.741737
H	2.365709	4.308486	-2.054882
H	3.871721	3.613123	-1.402046
H	3.131434	-4.203306	-1.315747
H	4.495108	-3.144826	-0.871011
H	3.485653	-2.745393	-2.276726
H	3.329916	0.118905	-1.513290
H	4.281083	0.395725	0.563416
H	7.233384	1.155031	5.080241
H	7.562647	0.419379	3.511441
H	6.761151	1.998469	3.602216
H	5.623121	-1.601299	5.816449
H	4.295518	-2.079065	4.759847
H	5.971097	-2.184386	4.189251
H	4.124498	1.309734	6.070933
H	3.769013	2.174684	4.573632
H	2.884610	0.690955	4.981567

TS3c

C	-1.216148	3.139342	0.046617
C	-0.051291	3.884032	0.012982
C	1.173581	3.211390	0.013704
C	1.267518	1.817799	0.048224
C	0.100906	1.075449	0.081963
C	-1.171392	1.717138	0.138311
O	0.169957	-0.286021	0.061847
P	-1.277909	-1.117381	0.266847
C	-0.926593	-2.146699	1.722427
Ir	-2.788136	0.688137	0.344822
P	-3.788990	2.816824	0.198737
C	-4.602499	3.606858	1.618425
O	-2.421903	3.774387	-0.005005
C	-1.339139	-2.222171	-1.166922
C	-4.784843	3.194919	-1.267413
H	-1.542854	-1.638386	-2.064574
H	-0.395631	-2.761872	-1.269199
H	-0.771039	-1.512651	2.595832
H	-0.037349	-2.756103	1.548031
H	-1.781544	-2.801123	1.910110
H	-2.153816	-2.937687	-1.030107
H	-4.880844	4.275303	-1.391978
H	-4.315103	2.753713	-2.146826
H	-5.778487	2.755249	-1.149474
H	-4.779781	4.664502	1.412501
H	-3.976162	3.505935	2.505392
H	-5.557765	3.109453	1.804006
H	-5.150433	-0.422739	0.248230
H	-0.104247	4.963924	-0.024796
H	2.086799	3.793639	-0.020554
H	2.224979	1.314283	0.036315
O	-3.802468	-0.026477	-1.248806
C	-4.968305	-0.527745	-0.859296
O	-5.766545	-1.049084	-1.580915

IM4c

C	-0.978537	1.845216	0.121245
C	-1.127453	3.264036	-0.058184
C	-0.033139	4.076539	-0.313134
C	1.223516	3.482168	-0.431666
C	1.432684	2.103256	-0.298233
C	0.341575	1.300077	-0.035415
O	-2.351431	3.814028	0.039829
P	-3.660334	2.723033	0.252070
C	-4.519505	3.019302	-1.329071
O	0.518060	-0.040553	0.094028
P	-0.854879	-0.934250	0.517698
C	-1.021342	-2.103271	-0.863432
Ir	-2.487060	0.729030	0.647271
O	-3.871879	-0.551388	-0.253248
C	-5.132854	-0.240516	-0.284282
O	-5.658933	0.747140	0.209242
C	-0.178325	-1.912034	1.898325
C	-4.616632	3.611691	1.515008
H	-1.237531	-1.549167	-1.776671
H	-0.110945	-2.692080	-0.988508
H	0.042218	-1.259499	2.743172
H	0.737935	-2.414305	1.581202
H	-0.909730	-2.663309	2.203893
H	-1.868243	-2.761714	-0.658374
H	-4.521396	4.092611	-1.528524
H	-3.993075	2.502812	-2.132404
H	-5.538007	2.642101	-1.263794
H	-4.808693	4.634203	1.184597
H	-4.062571	3.631571	2.453474
H	-5.560808	3.084776	1.655257
H	-5.720345	-0.999288	-0.826952
H	-0.164705	5.144382	-0.423871
H	2.078543	4.116017	-0.638376
H	2.419181	1.671097	-0.397354
H	-3.485516	0.257909	2.025500
Si	-4.099705	-0.470803	3.296439
C	-2.791190	-0.247775	4.615388
C	-5.670905	0.480120	3.634560
C	-4.396867	-2.251038	2.809364
H	-4.868586	-2.774939	3.647796
H	-5.066209	-2.332210	1.951132
H	-3.470522	-2.778004	2.571989

H	-6.206190	0.003693	4.463223
H	-5.466367	1.512897	3.925141
H	-6.322752	0.484993	2.759378
H	-3.183104	-0.606651	5.573378
H	-1.881053	-0.811400	4.400771
H	-2.523117	0.803660	4.743687

TS5c

C	-1.075283	1.761505	0.289134
C	-1.213700	3.179226	0.142438
C	-0.119282	3.993540	-0.114173
C	1.138982	3.403621	-0.229436
C	1.339399	2.022604	-0.101023
C	0.242969	1.226079	0.160225
O	-2.440310	3.730803	0.272022
P	-3.773179	2.645638	0.228448
C	-4.360204	3.028684	-1.461518
O	0.419998	-0.123163	0.322126
P	-0.980699	-1.042708	0.456624
C	-1.012886	-1.980504	-1.106910
Ir	-2.640917	0.610021	0.580325
O	-4.314526	-0.710022	0.176794
C	-5.472663	-0.198008	-0.060377
O	-5.787431	0.987860	0.046514
C	-0.440666	-2.251172	1.706266
C	-4.858314	3.539976	1.380656
H	-1.087211	-1.287665	-1.946003
H	-0.114499	-2.591009	-1.215632
H	-0.376799	-1.782466	2.687935
H	0.544945	-2.630448	1.428021
H	-1.147145	-3.082052	1.740891
H	-1.900809	-2.617190	-1.108543
H	-4.397140	4.113372	-1.577887
H	-3.662983	2.615075	-2.191147
H	-5.345285	2.593980	-1.611360
H	-4.896624	4.587773	1.076774
H	-4.450569	3.482063	2.389715
H	-5.853564	3.102270	1.348669
H	-6.221373	-0.941799	-0.376703
H	-0.253001	5.061585	-0.222109
H	1.995478	4.036077	-0.432566
H	2.323699	1.584827	-0.199182
H	-3.000331	0.572969	2.158772
Si	-3.834233	-0.374533	3.296011
C	-2.614034	0.067542	4.646219
C	-5.470097	0.499273	3.469562
C	-3.953467	-2.195309	2.930118
H	-4.708200	-2.622644	3.600305
H	-4.274512	-2.367953	1.902664
H	-3.014357	-2.716787	3.118033
H	-6.029691	-0.006255	4.265852
H	-5.340469	1.540452	3.769838
H	-6.071893	0.470879	2.561509
H	-2.987632	-0.346838	5.590512
H	-1.621789	-0.352469	4.470915
H	-2.514425	1.147464	4.773246

IM5c

C	-1.163393	1.728497	0.513953
C	-1.320606	3.118989	0.369770
C	-0.229524	3.933867	0.096530
C	1.032753	3.350218	-0.011963
C	1.226466	1.974663	0.130733
C	0.124871	1.179501	0.404551
O	-2.571579	3.664176	0.506300
P	-3.880880	2.602809	0.346316
C	-4.316022	2.877341	-1.409618
O	0.288338	-0.182039	0.578085
P	-1.096600	-1.113627	0.568696
C	-1.143029	-1.853477	-1.097142
Ir	-2.787913	0.526368	0.755183
O	-4.243999	-0.734671	0.003664
C	-5.465369	-0.324941	-0.245064
O	-5.917822	0.777839	0.006243
C	-0.578897	-2.466204	1.666428
C	-5.109858	3.510674	1.323759
H	-1.172480	-1.063202	-1.848663
H	-0.266501	-2.481841	-1.265597
H	-0.512807	-2.126583	2.699135
H	0.404388	-2.814114	1.341966
H	-1.293383	-3.287632	1.595470
H	-2.055235	-2.448170	-1.185804
H	-4.360970	3.953693	-1.587218
H	-3.549124	2.444690	-2.053683
H	-5.278839	2.420446	-1.627620
H	-5.194997	4.518668	0.912509
H	-4.786968	3.578239	2.361389
H	-6.066817	2.996854	1.258714
H	-6.075949	-1.105961	-0.721525
H	-0.372612	4.999072	-0.028664
H	1.887776	3.981585	-0.221110
H	2.205935	1.526113	0.030229
H	-2.046077	1.231178	1.940875
Si	-3.622433	-0.232817	3.027263
C	-2.308666	0.189233	4.306540
C	-5.216662	0.673433	3.418276
C	-3.935803	-2.084294	2.940650
H	-4.609178	-2.343727	3.765709
H	-4.418823	-2.375350	2.007757
H	-3.027865	-2.674370	3.068397
H	-5.627944	0.209092	4.323269
H	-5.054169	1.727863	3.643598
H	-5.966751	0.591764	2.630852
H	-2.645134	-0.201024	5.274599
H	-1.333717	-0.256266	4.097474
H	-2.171858	1.267609	4.421356

TS6c

C	-1.030276	1.825171	0.293637
C	-1.125907	3.222803	0.179577
C	-0.030908	4.008106	-0.153075
C	1.199676	3.384384	-0.357208
C	1.347258	2.002609	-0.236863
C	0.232619	1.248266	0.097812
O	-2.345603	3.824325	0.412000
P	-3.675596	2.806159	0.365693
C	-4.296000	3.127807	-1.322703
O	0.373826	-0.119233	0.263242
P	-1.005709	-0.986541	0.591569
C	-1.207777	-2.040877	-0.884347
Ir	-2.655682	0.704051	0.736266
O	-4.374502	-0.589306	0.682655
C	-5.557807	-0.165638	0.217779
O	-5.887833	0.983523	0.074157
C	-0.365553	-2.102522	1.881448
C	-4.792158	3.675868	1.502170
H	-1.387987	-1.414960	-1.759737
H	-0.309980	-2.639158	-1.051410
H	-0.162415	-1.541843	2.793679
H	0.567656	-2.542237	1.522842
H	-1.078838	-2.898314	2.096576

H	-2.067875	-2.699776	-0.743682
H	-4.408540	4.206711	-1.449229
H	-3.568437	2.762865	-2.049360
H	-5.251285	2.631852	-1.478211
H	-4.834567	4.729478	1.218399
H	-4.418783	3.597793	2.523328
H	-5.785523	3.234350	1.431544
H	-6.214024	-1.013386	-0.025759
H	-0.145215	5.079955	-0.247061
H	2.062100	3.987267	-0.614286
H	2.302081	1.517892	-0.392052
H	-2.069585	1.307327	2.020011
Si	-3.957851	-0.549842	2.829911
C	-2.741695	0.060458	4.148120
C	-5.626293	0.174385	3.274548
C	-3.955652	-2.424487	2.771930
H	-4.967403	-2.789970	2.581233
H	-3.321848	-2.827444	1.982202
H	-3.616205	-2.834005	3.727733
H	-5.765205	-0.005441	4.346892
H	-5.689545	1.252462	3.121078
H	-6.460045	-0.295189	2.751134
H	-3.081499	-0.387564	5.091720
H	-1.708246	-0.248530	3.992518
H	-2.766091	1.144563	4.274144

TS6a

Si	-4.086440	-0.893718	1.749050
H	-2.368514	-1.082305	1.108791
Ir	1.013143	0.021923	-0.008812
O	-1.136923	0.316316	0.201382
C	-2.077220	-0.562583	0.062711
O	-3.374017	-0.032854	-0.282181
Si	-3.668133	0.943050	-1.685622
C	-2.683514	0.207178	-3.101524
H	-1.607486	0.243421	-2.912377
H	-2.966930	-0.830937	-3.297656
H	-2.872905	0.772975	-4.018973
C	-5.511937	0.801380	-1.974288
H	-5.814349	-0.225934	-2.193223
H	-5.798785	1.415522	-2.833626
H	-6.094636	1.157232	-1.120079
C	-3.163067	2.708939	-1.327559
H	-2.097655	2.758787	-1.094655
H	-3.356505	3.348830	-2.194010
H	-3.716186	3.123560	-0.480496
H	-1.861031	-1.436237	-0.576537
H	1.111499	0.390003	-1.498480
P	1.316218	-2.205820	-0.613968
C	0.917454	-2.775462	-2.300012
C	3.023308	-0.093586	0.068801
H	1.319317	-2.064259	-3.022154
H	1.356360	-3.758833	-2.480590
H	-0.165697	-2.832601	-2.428709
C	0.767311	-3.573718	0.468702
H	1.240115	-4.509270	0.162636
H	1.047541	-3.356175	1.500456
H	-0.318160	-3.683682	0.410284
O	2.967088	-2.404944	-0.565274
C	3.709737	-1.284995	-0.199503
C	5.090848	-1.392333	-0.122282
H	5.575260	-2.336684	-0.333797
C	5.823612	-0.257639	0.226234
H	6.903000	-0.321578	0.290888
C	5.194071	0.959030	0.488274
H	5.758916	1.843936	0.751276
C	3.810545	1.019136	0.398434
O	3.177527	2.237188	0.638087
P	1.519178	2.233693	0.530614
C	1.019161	2.909220	2.150149
H	1.511697	3.866588	2.332566
H	-0.064405	3.046736	2.162753
H	1.296507	2.208980	2.939328
C	1.198153	3.580686	-0.652392
H	1.704663	4.493316	-0.331678
H	1.565284	3.291123	-1.637252
H	0.123431	3.764073	-0.714184
C	-5.386004	-1.867123	0.841349
H	-5.949625	-1.252356	0.141012
H	-6.075952	-2.297827	1.575416
H	-4.941870	-2.699819	0.289473
C	-4.382763	0.862139	2.283210
H	-3.437315	1.407958	2.336503
H	-5.044761	1.392673	1.598767
H	-4.826445	0.865786	3.284126
C	-3.403555	-1.914853	3.177279
H	-4.199753	-2.026444	3.924555
H	-2.556310	-1.432417	3.670308
H	-3.103442	-2.919107	2.868575

IM8

Si	-4.994571	-1.356975	0.996517
H	-2.567893	-0.016354	-0.544365
Ir	1.593428	0.014591	-0.086078
O	-0.626551	-0.085749	-0.016799
C	-1.514679	0.093587	-0.837053
O	-4.709703	-0.058974	-0.037044
Si	-5.510875	1.219497	-0.781616
C	-6.421500	0.589095	-2.296854
H	-5.741810	0.094481	-2.997102
H	-7.201698	-0.128470	-2.028270
H	-6.904411	1.412770	-2.831952
C	-6.696649	2.032824	0.426045
H	-7.489042	1.348273	0.742530
H	-7.183234	2.897264	-0.036389
H	-6.180381	2.385505	1.323439
C	-4.174843	2.446428	-1.297330
H	-3.516850	2.031471	-2.067853
H	-4.624621	3.350387	-1.718984
H	-3.564853	2.755169	-0.442403
H	-1.274961	0.369643	-1.875060
H	1.646659	0.304994	-1.597595
P	2.055383	-2.241686	-0.502154
C	1.742164	-2.981591	-2.134969
C	3.585685	0.044567	0.089647
H	2.130630	-2.323624	-2.912658
H	2.232852	-3.954439	-2.206145
H	0.666829	-3.109402	-2.279649
C	1.515750	-3.497315	0.704370
H	1.994517	-4.455846	0.493414
H	1.785062	-3.177060	1.711821
H	0.430569	-3.612017	0.650413
O	3.710876	-2.309617	-0.384676
C	4.355083	-1.120319	-0.062705
C	5.734005	-1.125989	0.085141
H	6.287589	-2.048017	-0.034389
C	6.374362	0.077237	0.381513
H	7.451035	0.088849	0.499134
C	5.660023	1.266788	0.524697
H	6.156086	2.202464	0.746708
C	4.282438	1.231279	0.369220
O	3.563744	2.416121	0.484274

P	1.912718	2.289444	0.355271
C	1.335031	2.978219	1.941874
H	1.754231	3.974528	2.097174
H	0.244193	3.038697	1.939300
H	1.648322	2.327367	2.759436
C	1.514401	3.555630	-0.889422
H	1.940222	4.517646	-0.597023
H	1.925209	3.257377	-1.854269
H	0.429957	3.654250	-0.978671
C	-5.117842	-0.730668	2.762760
H	-4.220205	-0.177390	3.054570
H	-5.234981	-1.563509	3.463262
H	-5.976408	-0.067220	2.898045
C	-3.507874	-2.502427	0.825852
H	-2.580834	-2.006025	1.129531
H	-3.630296	-3.383463	1.463178
H	-3.392957	-2.856815	-0.203360
C	-6.564531	-2.255145	0.492891
H	-6.728388	-3.129056	1.131201
H	-6.513908	-2.607382	-0.541252
H	-7.448540	-1.617653	0.586792

TS8b

C	4.264360	1.195673	-0.742900
C	3.593029	0.000727	-0.443125
C	4.256781	-1.200641	-0.733918
C	5.517200	-1.229850	-1.319148
C	6.134174	-0.011711	-1.610191
C	5.525000	1.212420	-1.328218
Ir	1.710308	0.010044	0.391903
P	2.135637	-2.252477	0.337899
C	2.423968	-3.146345	1.907061
O	3.637920	-2.395579	-0.412301
O	3.653226	2.396821	-0.430586
P	2.150077	2.269096	0.320545
C	2.443722	3.174687	1.880860
C	1.200521	3.456347	-0.698355
C	1.180815	-3.444347	-0.669045
C	-1.647403	-0.167398	-1.660686
Si	-2.335711	-0.028655	0.070056
O	-4.186327	-0.058146	-0.281028
C	-2.291386	-1.531831	1.175687
C	-2.307559	1.638992	0.899059
C	-4.833694	-0.388456	-1.303190
H	0.088319	0.018971	0.642275
H	1.338936	0.018474	1.905542
H	1.047048	-3.042405	-1.675649
H	1.711144	-4.398264	-0.734901
H	3.096479	-2.557926	2.531827
H	2.866955	-4.125718	1.711687
H	1.477031	-3.269519	2.432930
H	0.201229	-3.605318	-0.214267
H	1.735191	4.402777	-0.779703
H	1.056377	3.038207	-1.696891
H	0.223798	3.629605	-0.242128
H	2.888882	4.149148	1.678884
H	3.113156	2.588278	2.508013
H	1.495449	3.303282	2.407807
H	6.017242	2.151008	-1.546886
H	7.118511	-0.016533	-2.066647
H	6.003452	-2.173136	-1.530902
H	-3.046523	-1.446382	1.964585
H	-2.493165	-2.444715	0.606531
H	-1.310895	-1.622444	1.644543
H	-3.089338	1.698668	1.663945
H	-1.341942	1.802657	1.380824
H	-2.477026	2.444599	0.175773
H	-2.028719	0.620143	-2.318147
H	-0.559699	-0.063744	-1.610861
H	-1.861237	-1.141092	-2.115293
O	-6.095725	-0.382821	-1.368352
Si	-7.363557	0.074295	-0.154013
C	-8.908551	-0.313212	-1.111665
C	-7.083380	-1.026573	1.323085
C	-7.092060	1.890375	0.171092
H	-7.886894	-0.865565	2.050434
H	-7.099806	-2.082922	1.049748
H	-6.138689	-0.810853	1.825811
H	-7.868517	2.255788	0.849954
H	-6.126340	2.080701	0.645076
H	-7.152975	2.479572	-0.744923
H	-9.792200	-0.078581	-0.509922
H	-8.967486	0.271753	-2.032009
H	-8.959976	-1.373079	-1.375730
H	-4.314664	-0.703800	-2.215107

TS8b-syn

Ir	-1.068580	0.510431	0.025175
Si	1.604365	2.770781	0.137844
C	3.458570	3.221381	0.149233
C	-2.353747	-1.077445	0.013043
C	-2.909447	-1.562968	-1.178571
C	-3.755818	-2.662801	-1.215196
C	-4.068184	-3.294941	-0.010802
C	-3.555858	-2.843268	1.206045
C	-2.713367	-1.739551	1.195025
O	-2.603647	-0.908414	-2.365863
P	-1.587416	0.412480	-2.232524
C	-2.583903	1.737465	-2.991364
O	-2.212175	-1.263503	2.399984
P	-1.216962	0.076994	2.302472
C	0.228433	-0.502704	3.258340
O	2.132400	0.816595	0.348075
C	3.107776	0.197182	-0.102040
O	3.296175	-1.040079	0.180021
C	0.963752	3.453716	1.765080
C	1.023837	3.540772	-1.484809
C	-0.344645	0.001068	-3.510880
C	-2.060304	1.249356	3.415297
H	-4.163227	-3.007413	-2.156772
H	-3.808321	-3.327800	2.140269
H	-4.729101	-4.153477	-0.020261
H	0.110105	1.774829	0.022553
H	-2.095363	1.645405	0.180299
H	-3.433405	1.960328	-2.345428
H	-1.976663	2.638508	-3.099317
H	-2.947715	1.422353	-3.971301
H	0.245829	-0.855141	-3.181790
H	-0.840762	-0.250303	-4.450571
H	0.317038	0.855259	-3.669316
H	-0.097565	-0.926534	4.210332
H	0.905144	0.333700	3.443213
H	0.760510	-1.262663	2.685511
H	1.196689	2.767562	2.584174
H	1.458165	4.403533	1.989769
H	-0.115485	3.606056	1.739119
H	1.226344	2.870385	-2.325828
H	1.564983	4.472522	-1.675047
H	-0.048630	3.738303	-1.461743
H	4.003482	2.836310	1.016049
H	4.000889	2.932386	-0.757315
H	3.518861	4.314084	0.196469

H	-1.436916	2.133721	3.560362
H	-3.005508	1.553903	2.965216
H	-2.255414	0.777386	4.380271
H	3.831277	0.691639	-0.759195
Si	4.593346	-2.182203	-0.257171
C	5.267714	-2.716186	1.392392
C	3.697053	-3.519679	-1.190572
C	5.826537	-1.238834	-1.298810
H	6.048311	-3.470207	1.251499
H	5.711467	-1.880953	1.940364
H	4.488664	-3.160262	2.016930
H	4.392508	-4.322663	-1.453731
H	2.898042	-3.959572	-0.588588
H	3.260095	-3.146163	-2.120343
H	6.644548	-1.914398	-1.569357
H	5.400306	-0.870131	-2.236010
H	6.276797	-0.398022	-0.763778

(POCOP)IrH2 +[HC(OSiMe3)2]$^{+}$

C	4.843527	-0.735025	2.594483
C	3.975060	-0.884373	1.519442
C	3.386894	0.214590	0.875193
C	3.728025	1.494384	1.338147
C	4.591788	1.689114	2.409455
C	5.135735	0.560346	3.025754
Ir	2.067824	-0.041559	-0.691993
P	2.678545	-2.221973	-0.295208
C	1.544133	-3.554446	0.241606
O	3.682926	-2.156054	1.060967
O	3.188233	2.596107	0.698440
P	2.225110	2.248956	-0.644904
C	3.155041	3.074894	-1.984407
C	0.841154	3.403071	-0.318069
C	3.762850	-3.047509	-1.512191
C	-1.458604	-0.152247	1.247516
Si	-2.378871	-0.476590	-0.327293
O	-4.099514	-0.662990	0.287293
C	-2.448434	0.953967	-1.523062
C	-2.100311	-2.129649	-1.125205
C	-5.175199	-0.073179	0.026942
O	-6.247357	-0.401558	0.609197
Si	-7.950613	0.191073	0.518840
C	-8.886154	-1.348033	0.065687
C	-8.250489	0.817682	2.242071
C	-7.972731	1.518372	-0.794751
H	5.282743	-1.600855	3.072694
H	5.812914	0.694435	3.861711
H	4.837561	2.688164	2.745271
H	1.222569	4.406684	-0.118992
H	0.182152	3.431199	-1.188708
H	0.277869	3.055955	0.548504
H	4.084068	2.531204	-2.155613
H	2.563920	3.049594	-2.902496
H	3.379960	4.109542	-1.717885
H	4.567071	-2.364849	-1.786427
H	3.185188	-3.282030	-2.408948
H	4.185232	-3.963605	-1.094700
H	2.112481	-4.417113	0.595295
H	0.914954	-3.854410	-0.599180
H	0.911007	-3.186641	1.049346
H	0.635793	-0.243314	-1.424297
H	2.043616	-0.170046	-2.263407
H	-1.074544	-2.160227	-1.503019
H	-2.775313	-2.296696	-1.968516
H	-2.227481	-2.946902	-0.411387
H	-3.093895	0.762040	-2.384949
H	-1.433764	1.096641	-1.908051
H	-2.750702	1.893527	-1.051909
H	-0.390954	-0.068451	1.007343
H	-1.775759	0.778362	1.725261
H	-1.587445	-0.968186	1.962832
H	-5.189146	0.735627	-0.712724
H	-9.959374	-1.134875	0.037843
H	-8.600346	-1.728100	-0.918351
H	-8.725650	-2.140782	0.800379
H	-8.997189	1.893625	-0.889404
H	-7.346982	2.379446	-0.543800
H	-7.684051	1.146444	-1.781886
H	-9.287281	1.154610	2.339653
H	-8.086919	0.032467	2.984156
H	-7.604791	1.664278	2.488984

IM9b

Ir	0.671412	-1.132972	0.264222
Si	-4.286542	-1.201268	0.004911
C	-6.095765	-0.816398	-0.170949
C	2.200595	0.227080	0.012924
C	2.912759	0.304920	-1.195186
C	3.946936	1.215114	-1.385222
C	4.279442	2.060676	-0.324874
C	3.614689	2.012749	0.903314
C	2.587026	1.088650	1.051540
O	2.566075	-0.547003	-2.225509
P	1.364303	-1.679813	-1.862943
C	2.267573	-3.252651	-2.074167
O	1.913224	1.012860	2.258862
P	0.845379	-0.293552	2.401286
C	-0.466191	0.502018	3.398144
O	-3.578966	0.454284	-0.081052
C	-2.448168	0.985943	-0.274724
O	-2.380631	2.245768	-0.335831
C	-3.798429	-1.849007	1.680353
C	-3.564440	-2.141683	-1.432727
C	0.352612	-1.554091	-3.381101
C	1.704836	-1.355546	3.615702
H	4.482201	1.253127	-2.325030
H	3.898409	2.660513	1.722636
H	5.088917	2.770013	-0.454884
H	-0.852778	-1.649512	0.234687
H	0.292528	-2.612000	0.692849
H	3.005082	-3.349800	-1.277421
H	1.563656	-4.084330	-1.999526
H	2.769229	-3.279889	-3.043430
H	-0.151656	-0.587911	-3.408685
H	0.984017	-1.654080	-4.266278
H	-0.397521	-2.347881	-3.380132
H	-0.030262	1.001645	4.265670
H	-1.168298	-0.262534	3.737782
H	-1.004300	1.236433	2.798867
H	-4.151814	-1.194435	2.481260
H	-4.251292	-2.833319	1.837166
H	-2.715417	-1.965229	1.764248
H	-3.794086	-1.661941	-2.387971
H	-3.998748	-3.146381	-1.460269
H	-2.481784	-2.253611	-1.338190
H	-6.445557	-0.162295	0.631428
H	-6.313675	-0.332091	-1.126030
H	-6.681703	-1.739728	-0.126303
H	1.055973	-2.192580	3.882405
H	2.612644	-1.752928	3.162021
H	1.960620	-0.786547	4.511663

H	-1.539985	0.360220	-0.356707
Si	-1.165864	3.523256	-0.728126
C	-2.276654	4.864411	-1.378515
C	-0.361666	3.926768	0.900422
C	-0.025726	2.788451	-2.001451
H	-1.682834	5.742723	-1.650121
H	-2.818398	4.542730	-2.271435
H	-3.007662	5.176535	-0.628755
H	0.296594	4.791404	0.765997
H	-1.101709	4.197027	1.658137
H	0.255512	3.109506	1.280283
H	0.692719	3.551785	-2.317535
H	0.551845	1.952107	-1.600900
H	-0.564927	2.463076	-2.895404

TS10b

Ir	-0.694178	-0.243575	0.160426
Si	3.833796	-2.067146	0.584595
C	5.631140	-2.186813	0.112906
C	-2.708626	-0.146308	-0.183665
C	-3.607390	0.238045	0.824789
C	-4.970333	0.366413	0.595632
C	-5.451074	0.087699	-0.685237
C	-4.604944	-0.315525	-1.719814
C	-3.248358	-0.431631	-1.448802
O	-3.110525	0.485938	2.093889
P	-1.453759	0.292471	2.278806
C	-1.372750	-0.969271	3.595134
O	-2.394684	-0.853802	-2.454764
P	-0.777114	-0.991556	-2.029690
C	0.014360	-0.108091	-3.417873
O	3.256283	-0.688537	-0.361880
C	2.724167	0.421344	0.004942
O	2.642198	1.356600	-0.872406
C	2.809438	-3.507010	-0.001419
C	3.565132	-1.653608	2.389239
C	-1.045188	1.851759	3.142510
C	-0.451264	-2.751416	-2.383312
H	-5.634909	0.667378	1.394882
H	-4.987599	-0.540381	-2.706758
H	-6.512777	0.182290	-0.880741
H	0.981572	-0.044235	0.311672
H	-0.183948	-1.613887	0.660959
H	-1.722070	-1.922781	3.198750
H	-0.340068	-1.083081	3.932033
H	-2.000141	-0.675159	4.438761
H	-1.164451	2.692559	2.458501
H	-1.706853	1.993431	3.999547
H	-0.009775	1.815661	3.488743
H	-0.294452	-0.540864	-4.371478
H	1.099509	-0.176147	-3.314604
H	-0.278519	0.941477	-3.390734
H	2.949185	-3.682647	-1.071089
H	3.110873	-4.419153	0.522762
H	1.746220	-3.346647	0.190189
H	4.147136	-0.787149	2.715980
H	3.891006	-2.501052	3.000694
H	2.510538	-1.477365	2.617102
H	5.751476	-2.304818	-0.966926
H	6.193793	-1.302358	0.422715
H	6.089824	-3.056134	0.594291
H	0.619214	-2.947523	-2.298463
H	-0.984046	-3.369457	-1.660517
H	-0.788814	-2.999641	-3.391388
H	2.691924	0.674724	1.062135
Si	2.287488	3.079805	-0.696295

C	3.577421	3.878654	-1.773387
C	0.543520	3.298790	-1.316114
C	2.476509	3.498010	
1.117698			
H	3.429473	4.962711	-1.798777
H	4.588247	3.690079	-1.403128
H	3.518398	3.512363	-2.801372
H	0.204332	4.324019	-1.138500
H	0.475188	3.111722	-2.390116
H	-0.147243	2.622028	-0.803829
H	2.303191	4.570337	1.253838
H	1.751989	2.975892	1.748069
H	3.482624	3.290321	1.493027

IM10

Ir	0.797426	-0.002000	-0.300473
Si	-4.403979	-0.934162	-1.192957
C	-4.351012	-2.493967	-2.220580
C	2.673186	-0.563347	0.118840
C	3.771402	0.208261	-0.279885
C	5.073962	-0.155052	0.031446
C	5.281650	-1.334934	0.745573
C	4.218359	-2.148009	1.136985
C	2.927436	-1.756550	0.809510
O	3.548604	1.366330	-1.013590
P	1.965010	1.713347	-1.395195
C	1.972312	1.734337	-3.216164
O	1.863228	-2.571666	1.171840
P	0.344854	-2.050780	0.728009
C	-0.585827	-2.173602	2.286189
O	-2.749436	-0.652502	-0.804829
C	-2.203055	0.519994	-0.368526
O	-2.400370	0.709085	0.987372
C	-5.038101	0.536578	-2.172683
C	-5.372737	-1.169975	0.391854
C	1.855344	3.469448	-0.917218
C	-0.292651	-3.393922	-0.316248
H	5.898920	0.469561	-0.285405
H	4.379744	-3.070492	1.679303
H	6.293205	-1.630895	0.995120
H	-1.071640	0.453138	-0.615604
H	0.795830	-0.764151	-1.640165
H	2.176156	0.730622	-3.589821
H	0.992850	2.052791	-3.580898
H	2.737416	2.420905	-3.583840
H	1.934590	3.566091	0.165955
H	2.664882	4.033020	-1.385824
H	0.896537	3.879129	-1.243516
H	-0.576012	-3.203000	2.650062
H	-1.612954	-1.849002	2.108060
H	-0.131925	-1.526383	3.037031
H	-4.440246	0.718379	-3.070362
H	-6.064008	0.342785	-2.501093
H	-5.064296	1.458740	-1.584828
H	-4.988121	-2.014399	0.970341
H	-6.425817	-1.372158	0.172890
H	-5.326917	-0.281410	1.026140
H	-3.745825	-2.361850	-3.121189
H	-3.939622	-3.334345	-1.654428
H	-5.359008	-2.778844	-2.536530
H	-1.306257	-3.132150	-0.627697
H	0.334531	-3.493121	-1.202521
H	-0.300707	-4.337623	0.232456
H	-2.538129	1.389579	-0.951959
Si	-1.893930	1.994300	1.979894
C	-2.882366	1.820221	3.550266

C	-0.048075	1.739612	2.307012
C	-2.196472	3.628135	1.109340
H	-2.569299	2.560530	4.292495
H	-3.949221	1.968105	3.364256
H	-2.753930	0.829411	3.993495
H	0.401810	2.603561	2.806231
H	0.132812	0.867230	2.937018
H	0.521008	1.618054	1.364942
H	-1.908127	4.461655	1.757161
H	-1.628022	3.729593	0.180076
H	-3.255625	3.757175	0.869158

TS8bi

Ir	-1.477398	0.060432	0.407925
H	-1.069796	0.133458	1.910214
P	-1.894360	2.318554	0.237826
C	-0.938433	3.448161	-0.840145
C	-2.167809	3.296700	1.756054
O	-3.399755	2.429613	-0.508991
C	-4.026452	1.222871	-0.766035
C	-5.284581	1.227261	-1.354609
C	-5.905976	-0.002022	-1.582990
C	-5.304339	-1.212613	-1.234161
C	-4.045545	-1.171146	-0.648021
C	-3.368064	0.035372	-0.409223
O	-3.438291	-2.356458	-0.273308
P	-1.932092	-2.197728	0.463945
H	-0.855186	-3.101358	-1.507456
C	-0.996891	-3.452061	-0.484760
C	-2.230042	-3.002681	2.076109
H	0.154127	0.049403	0.606850
C	1.936140	-0.378181	-1.691867
Si	2.678917	-0.219630	0.005251
C	2.732597	1.528048	0.652133
C	2.539631	-1.523515	1.325858
O	4.660680	-0.501139	-0.484516
Si	6.104648	0.593873	-0.110366
C	6.248187	0.583162	1.746644
C	5.744001	2.238277	-0.906107
C	7.596298	-0.205641	-0.904648
C	4.874558	-1.751808	-1.085132
H	5.904885	-1.875383	-1.424938
O	4.006189	-2.549055	-1.194079
H	-5.800764	-2.158877	-1.405153
H	-6.888579	-0.016694	-2.040472
H	-5.765708	2.160021	-1.618349
H	-1.464714	4.397576	-0.958202
H	0.043357	3.629892	-0.397301
H	-0.807868	2.986959	-1.819615
H	-2.837985	2.747078	2.417081
H	-1.215311	3.445254	2.269655
H	-2.608491	4.265651	1.513306
H	-2.893283	-2.372648	2.668902
H	-1.282839	-3.112464	2.608652
H	-2.687507	-3.983819	1.934911
H	-1.542048	-4.398003	-0.502673
H	-0.019921	-3.605223	-0.021563
H	2.394827	-2.515423	0.898400
H	1.686850	-1.284364	1.963036
H	3.438284	-1.544050	1.948930
H	1.745754	1.698625	1.090911
H	2.870538	2.260697	-0.144005
H	3.470337	1.698715	1.436392
H	0.928501	0.042513	-1.652712
H	2.519845	0.182806	-2.427677
H	1.865717	-1.415248	-2.016764

H	7.124492	1.176461	2.029253
H	5.388446	1.011718	2.262997
H	6.408493	-0.429310	2.127060
H	8.445342	0.455798	-0.697409
H	7.857541	-1.181913	-0.489252
H	7.526144	-0.287270	-1.992443
H	6.687048	2.792071	-0.967038
H	5.375170	2.122980	-1.928798
H	5.040576	2.854518	-0.347376

(POCOP)IrH2 +[HCOO(SiMe3)2]$^{+}$

Ir	1.636157	0.072463	-0.708177
H	1.495822	0.162069	-2.273385
P	1.917400	2.329574	-0.379637
O	3.045710	2.454492	0.869909
C	3.577766	1.255238	1.308747
C	4.553932	1.269358	2.297828
C	5.085629	0.046074	
2.711036			
C	4.672789	-1.167934	2.158640
C	3.695052	-1.136404	1.171317
C	3.114590	0.063102	
0.731147			
O	3.282783	-2.323323	0.593512
P	2.155830	-2.166189	-0.653657
C	0.998653	-3.503031	-0.180384
C	3.072359	-2.874763	-2.066607
C	0.635314	3.463241	0.274127
C	2.717867	3.311342	-1.696010
H	0.140003	0.017132	-1.338181
Si	-2.798281	-0.332733	-0.064083
C	-2.771366	1.345916	-0.866476
C	-2.701312	-1.733456	-1.278077
C	-1.735524	-0.381953	1.453625
O	-4.001136	-2.458926	1.553421
C	-4.827665	-1.621135	1.456533
O	-4.639908	-0.485055	0.631422
Si	-6.110363	0.577503	0.181082
C	-7.563417	-0.139162	1.110299
C	-6.304882	0.349202	-1.655512
C	-5.715991	2.295242	0.775525
H	-5.793976	-1.596252	1.962924
H	5.103217	-2.108075	2.478121
H	5.849294	0.039320	3.480493
H	4.892751	2.204264	2.724877
H	1.083627	4.415777	0.564091
H	-0.120923	3.637360	-0.494483
H	0.164639	3.009948	1.147024
H	3.590429	2.769491	-2.060844
H	2.017845	3.440350	-2.524463
H	3.025068	4.287825	-1.316429
H	3.885242	-2.199574	-2.334009
H	2.400720	-2.966604	-2.922962
H	3.479361	-3.854762	-1.809784
H	1.543854	-4.432704	-0.005127
H	0.274141	-3.653455	-0.983670
H	0.470331	-3.221490	0.730835
H	-2.747228	-2.707200	-0.790294
H	-1.739059	-1.647778	-1.792961
H	-3.488514	-1.676299	-2.034175
H	-1.758916	1.416861	-1.283063
H	-2.881990	2.161790	-0.151247
H	-3.468583	1.484138	-1.692836
H	-0.709812	-0.189121	1.107417
H	-2.001546	0.404374	2.165248
H	-1.750104	-1.344092	1.963350
H	-7.202914	0.890365	-1.973240

H	-5.471980	0.735237	-2.243640
H	-6.455108	-0.702221	-1.914441
H	-8.433577	0.463249	0.824530
H	-7.801403	-1.171551	0.842843
H	-7.478581	-0.060394	2.197170
H	-6.647210	2.872290	0.767559
H	-5.348916	2.295225	1.805327
H	-4.999932	2.824515	0.147833

IM9bi

C	3.078393	-0.045311	0.257652
C	3.760333	-1.272776	0.226748
C	5.090802	-1.393852	0.607823
C	5.761581	-0.243027	1.024781
C	5.138278	1.005794	1.060194
C	3.807578	1.081981	0.670291
O	3.083914	-2.393863	-0.212757
P	1.479080	-2.146169	-0.663073
C	0.688578	-3.489906	0.291173
O	3.177583	2.313231	0.672132
P	1.572653	2.304795	0.165209
C	1.628568	3.517954	-1.200029
Ir	1.089599	0.096289	-0.270479
C	1.497238	-2.773986	-2.378985
C	0.814853	3.237177	1.546829
O	-2.580161	-2.417291	-0.410499
C	-2.612833	-1.299862	-0.793763
O	-3.451690	-0.337643	-0.140840
Si	-3.990214	1.173930	-1.095252
C	-3.313462	0.938775	-2.817813
Si	-3.934186	-0.837988	1.635890
C	-2.351120	-1.419772	2.417906
C	-5.291256	-2.088662	1.414482
C	-4.512334	0.751827	2.421097
C	-5.850216	1.081384	-1.074568
C	-3.216021	2.631288	-0.239604
H	0.454900	0.369813	-1.671786
H	-0.547333	0.157759	-0.163905
H	-2.069097	-0.855437	-1.622428
H	6.800294	-0.320210	1.324967
H	5.671595	1.893802	1.373292
H	5.587536	-2.354590	0.572033
H	1.207337	-4.433769	0.111475
H	-0.358010	-3.581646	-0.003621
H	0.740826	-3.249013	1.353504
H	1.364242	4.163683	1.725760
H	-0.224356	3.472151	1.306981
H	0.840063	2.625456	2.449167
H	2.040234	-2.070044	-3.009724
H	1.982514	-3.750997	-2.420770
H	0.473024	-2.862548	-2.748191
H	2.133666	4.430450	-0.877207
H	0.611836	3.756917	-1.519629
H	2.167126	3.082093	-2.041558
H	-6.237633	1.905352	-1.683913
H	-6.211005	0.152126	-1.523085
H	-6.291667	1.182524	-0.082510
H	-3.260828	3.491094	-0.916547
H	-3.715427	2.916192	0.686433
H	-2.162684	2.426540	-0.031153
H	-3.694372	1.772037	-3.419306
H	-2.222899	0.989338	-2.862209
H	-3.652310	0.021334	-3.305417
H	-5.638543	-2.412773	2.401406
H	-6.153125	-1.674185	0.885833
H	-4.939509	-2.970998	0.878050
H	-4.829859	0.483532	3.435932
H	-3.706857	1.480651	2.525360
H	-5.369052	1.226486	1.942584
H	-2.424409	-1.274601	3.500571
H	-2.157144	-2.474470	2.224583
H	-1.493569	-0.840476	2.061333

TS10bi

C	-3.018112	0.125204	-0.124480
C	-3.839294	-1.005174	0.001844
C	-5.214855	-0.949181	-0.178693
C	-5.790462	0.284880	-0.484643
C	-5.021893	1.444060	-0.604836
C	-3.650641	1.342309	-0.416054
O	-3.252357	-2.218306	0.334970
P	-1.586236	-2.210815	0.485575
C	-1.101663	-3.497752	-0.713778
O	-2.869767	2.487789	-0.503868
P	-1.226328	2.269086	-0.278280
C	-0.884150	3.483209	1.041293
Ir	-0.986945	-0.001684	0.106417
C	-1.358859	-2.994376	2.115284
C	-0.577350	3.060284	-1.795077
O	2.009305	-2.288947	0.265494
C	2.065250	-1.195110	0.726120
O	3.171023	-0.276178	0.167788
Si	3.827972	1.056199	1.213693
C	2.902805	0.972553	2.837418
Si	3.795422	-0.838858	-1.481486
C	2.297640	-1.200885	-2.530339
C	4.925405	-2.278988	-1.138407
C	4.720255	0.628044	-2.182111
C	5.634278	0.664264	1.468287
C	3.484662	2.669687	0.342603
H	-0.818956	0.249322	1.617994
H	0.724385	-0.172036	0.134973
H	1.818839	-0.845169	1.718688
H	-6.862748	0.345763	-0.628147
H	-5.474153	2.400703	-0.831674
H	-5.816424	-1.842885	-0.075921
H	-1.638933	-4.426847	-0.512780
H	-0.026032	-3.664451	-0.634262
H	-1.337769	-3.159552	-1.723708
H	-1.007158	4.056939	-1.914434
H	0.510454	3.137859	-1.737402
H	-0.840870	2.453712	-2.662646
H	-1.687587	-2.310145	2.897715
H	-1.942081	-3.915613	2.172261
H	-0.301564	-3.227768	2.257939
H	-1.291269	4.459534	0.771087
H	0.193534	3.568781	1.195649
H	-1.348208	3.143701	1.967653
H	6.056570	1.407525	2.153124
H	5.767570	-0.316149	1.933339
H	6.230169	0.694613	0.554993
H	3.622074	3.488466	1.056416
H	4.145468	2.859587	-0.503294
H	2.450830	2.707134	-0.008608
H	3.281181	1.792928	3.457423
H	1.825615	1.124752	2.734157
H	3.082533	0.051602	3.397720
H	5.332318	-2.647533	-2.086038
H	5.772922	-1.996911	-0.508599
H	4.392345	-3.101703	-0.660207
H	5.145573	0.291610	-3.134820
H	4.063169	1.469900	-2.408734

H	5.555111	0.982321	-1.576531
H	2.553286	-1.046828	-3.583173
H	1.949367	-2.225388	-2.403875
H	1.472424	-0.527973	-2.282373

IM10bi

C	2.100459	0.904599	0.825011
C	2.988323	0.262165	1.696241
C	3.556102	0.921900	2.777613
C	3.233754	2.262840	2.983687
C	2.362114	2.942677	2.133345
C	1.812566	2.253133	1.061549
O	3.302040	-1.073795	1.482266
P	2.646739	-1.787730	0.130767
C	1.997493	-3.362109	0.767342
O	0.938056	2.915715	0.209060
P	0.364541	2.055823	-1.094541
C	0.997945	3.004254	-2.515136
Ir	1.248763	-0.086246	-0.698139
C	4.101178	-2.248040	-0.858395
C	-1.417552	2.413389	-1.056050
O	1.288921	-2.569774	-2.914525
C	0.840892	-1.465488	-2.901277
O	-3.568458	-0.190135	0.437034
Si	-5.038000	0.205883	-0.281445
C	-4.822550	-0.006533	-2.136883
Si	-2.735882	-1.109892	1.533634
C	-0.898505	-0.653718	1.293529
C	-2.963682	-2.939786	1.184414
C	-3.220962	-0.679531	3.291790
C	-6.391369	-0.929392	0.356548
C	-5.419162	1.993203	0.146240
H	2.424964	0.324785	-1.616263
H	0.231107	-1.079494	-1.971770
H	0.784755	-0.797611	-3.773623
H	3.671512	2.789096	3.823018
H	2.111890	3.983615	2.291021
H	4.234704	0.393960	3.434773
H	2.787309	-3.912031	1.283376
H	1.634409	-3.953689	-0.076383
H	1.173715	-3.180491	1.456622
H	-1.572696	3.483041	-0.898551
H	-1.854612	2.125268	-2.015288
H	-1.910619	1.843636	-0.268696
H	4.631876	-1.347217	-1.167312
H	4.770637	-2.879206	-0.270668
H	3.772287	-2.792428	-1.745983
H	0.679268	4.046124	-2.442196
H	0.616126	2.577180	-3.445667
H	2.087295	2.960042	-2.526358
H	-7.352777	-0.664003	-0.094373
H	-6.197818	-1.977788	0.111946
H	-6.511998	-0.850996	1.441094
H	-6.360913	2.311450	-0.311439
H	-5.516605	2.131257	1.226852
H	-4.637674	2.672254	-0.207041
H	-5.743903	0.247695	-2.669621
H	-4.031420	0.640671	-2.527416
H	-4.570018	-1.039776	-2.392666
H	-2.368674	-3.553087	1.868448
H	-4.008639	-3.235156	1.314226
H	-2.674210	-3.199197	0.161656
H	-2.621550	-1.236936	4.017959
H	-3.086897	0.386520	3.494823
H	-4.270684	-0.924351	3.478745

H	-0.251157	-1.253614	1.935018
H	-0.655101	-0.858567	0.226996
H	-0.730199	0.397259	1.529323

TS11b

Ir	2.212555	-0.024842	0.203597
Si	-1.662930	0.704196	-0.078737
C	-1.281948	2.276485	0.861143
C	4.193662	-0.382152	-0.218719
C	5.126201	0.663416	-0.302036
C	6.459441	0.449104	-0.626096
C	6.874767	-0.862135	-0.865303
C	5.993933	-1.942030	-0.780966
C	4.669753	-1.681287	-0.453832
O	4.696598	1.954153	-0.035751
P	3.070829	2.111481	0.357303
C	2.595581	3.452142	-0.793234
O	3.782620	-2.740512	-0.340717
P	2.207693	-2.327754	0.074644
C	1.295876	-3.249281	-1.217400
C	-1.161801	0.588490	-1.880848
O	-3.570422	0.943495	-0.451577
C	-4.243385	-0.294902	-0.876314
O	-5.104592	-0.683550	0.117463
Si	-6.053090	-2.117937	0.183315
C	-6.818952	-2.059405	1.883145
C	-1.812069	-0.819535	1.004021
C	1.956650	-3.323745	1.584319
C	3.169190	2.936172	1.983218
Si	-4.642987	2.385744	-0.259013
C	-3.697218	3.859457	-0.916128
C	-6.139950	2.088529	-1.339689
C	-5.096574	2.502706	1.546244
C	-7.334668	-2.053064	-1.184609
C	-4.907734	-3.588298	-0.040009
H	0.572686	0.286348	0.247401
H	1.799041	-0.037970	1.693841
H	1.461811	-2.774795	-2.185222
H	1.642772	-4.283730	-1.261161
H	2.570447	-2.914265	2.386838
H	2.238466	-4.363268	1.405727
H	0.907811	-3.274793	1.884727
H	0.227184	-3.233438	-0.993186
H	3.302103	4.281707	-0.723176
H	2.593142	3.068318	-1.814014
H	1.592691	3.805956	-0.545454
H	3.818868	3.811738	1.927445
H	3.569810	2.233121	2.713760
H	2.169726	3.241788	2.300401
H	7.151615	1.279122	-0.682121
H	7.911952	-1.047616	-1.119337
H	6.327032	-2.956567	-0.956831
H	-2.805382	-0.886156	1.453246
H	-1.617705	-1.739546	0.448266
H	-1.069355	-0.756546	1.799279
H	-2.115992	2.613277	1.478434
H	-0.442121	2.065970	1.523170
H	-0.988483	3.089228	0.195068
H	-1.626044	1.392030	-2.459075
H	-0.079640	0.662835	-1.978986
H	-1.469435	-0.363356	-2.323077
H	-4.418534	4.668258	-1.075417
H	-2.933249	4.239426	-0.238989
H	-3.234910	3.651387	-1.884746
H	-5.809457	3.321043	1.690465
H	-5.572620	1.577108	1.876548

H	-4.241809	2.697850	2.197155
H	-6.794791	2.960082	-1.233940
H	-5.890594	2.007570	-2.401021
H	-6.715390	1.211848	-1.041119
H	-7.950807	-2.957507	-1.164553
H	-8.008622	-1.199695	-1.069339
H	-6.883986	-1.996945	-2.180130
H	-5.473878	-4.520081	0.054619
H	-4.433147	-3.605971	-1.025942
H	-4.121474	-3.606473	0.719738
H	-7.450425	-2.937167	2.049691
H	-6.055844	-2.047184	2.665642
H	-7.447821	-1.174061	2.008733
H	-4.752921	-0.069566	-1.816728
H	-3.449404	-1.023141	-1.057478

TS11b-syn

Ir	-1.418500	-0.009689	0.337537
Si	1.694730	-0.869758	1.919718
O	2.269456	-0.977972	-0.175855
C	2.166482	0.301022	-0.874661
O	2.908201	1.268616	-0.237404
Si	4.281024	2.159887	-0.755272
C	3.898471	3.946062	-0.350330
C	-3.205371	0.511387	-0.499658
C	-3.587639	1.853863	-0.648082
C	-4.775780	2.231331	-1.258656
C	-5.625777	1.229332	-1.726981
C	-5.306883	-0.121619	-1.587887
C	-4.108172	-0.453802	-0.972064
P	-2.321101	-2.115709	-0.081266
C	-2.829229	-3.194566	1.298061
P	-1.347754	2.307415	0.587848
C	-1.513951	3.073444	2.234479
O	-2.749574	2.839001	-0.151940
O	-3.792115	-1.792779	-0.805611
C	0.721862	-2.311057	2.646315
C	1.466871	0.791648	2.774255
C	3.511537	-1.277134	2.274776
C	-1.611006	-3.262649	-1.314493
C	-0.108426	3.335182	-0.273361
Si	2.958106	-2.351236	-1.043724
C	2.484115	-3.913441	-0.131555
C	2.196004	-2.367931	-2.759085
C	4.814548	-2.145682	-1.135661
C	5.774382	1.569177	
0.206811			
C	4.501992	1.918624	-2.605980
H	-6.557952	1.506556	-2.204559
H	-5.029114	3.279005	-1.354989
H	-5.971615	-0.899314	-1.940542
H	-2.326996	-4.055530	-1.540348
H	-0.690953	-3.704246	-0.929189
H	-1.388346	-2.714919	-2.231043
H	-0.350163	4.393613	-0.157387
H	0.884505	3.133370	0.131556
H	-0.115279	3.086352	-1.335855
H	-3.374638	-2.603299	2.034092
H	-3.476614	-3.990852	0.925538
H	-1.950833	-3.630997	1.776210
H	-1.792996	4.122449	2.116964
H	-0.572810	3.006209	2.781236
H	-2.291995	2.555918	2.796213
H	-1.808091	-0.144045	1.820657
H	0.195021	-0.464165	0.900977
H	0.431881	0.916290	3.091783
H	2.123829	0.841548	3.647945
H	-0.273255	-1.982195	2.948375
H	1.739245	1.608971	2.105005
H	0.609511	-3.136494	1.941702
H	3.621345	-1.289923	3.365732
H	4.184375	-0.512255	1.883949
H	3.847213	-2.249841	1.913042
H	1.255029	-2.699377	3.519884
H	2.891932	-4.758149	-0.697121
H	2.890135	-3.978820	0.878240
H	1.403348	-4.057521	-0.078101
H	2.446108	-3.318709	-3.241015
H	1.106080	-2.297827	-2.726029
H	2.568908	-1.576628	-3.413478
H	5.243745	-2.987287	-1.689171
H	5.106522	-1.233603	-1.661497
H	5.275717	-2.129761	-0.146269
H	6.650740	2.170379	-0.055509
H	5.619070	1.675387	1.284003
H	6.020211	0.524532	0.001989
H	4.754691	4.582343	-0.594482
H	3.040704	4.319448	-0.916027
H	3.689231	4.079345	0.714844
H	5.336980	2.538970	-2.946768
H	4.741423	0.889527	-2.888204
H	3.621129	2.232327	-3.173981
H	2.478627	0.143438	-1.909854
H	1.105762	0.561631	-0.838711

IM11b

C	5.723066	-1.628057	1.476168
C	4.713231	-1.282958	0.585569
C	4.035220	-0.057151	0.662389
C	4.429784	0.838690	1.667229
C	5.434448	0.534278	2.578446
C	6.065904	-0.706088	2.467256
Ir	2.516902	0.412412	-0.656294
P	3.187829	-1.617382	-1.493303
C	2.145829	-3.120602	-1.584261
O	4.365699	-2.170963	-0.416480
O	3.798922	2.067050	1.744805
P	2.653579	2.368429	0.540000
C	3.366540	3.838972	-0.278285
C	1.313775	3.066174	1.574916
C	4.099754	-1.641620	-3.077251
C	-0.892760	-1.154490	1.027116
Si	-2.032827	-0.545849	-0.307102
O	-3.745245	-1.113911	0.104154
Si	-4.338856	-2.068865	1.552753
C	-4.238126	-0.917570	3.012495
C	-2.145132	1.313498	-0.334176
C	-1.694540	-1.324354	-1.966690
C	-4.807583	-0.521020	-0.762634
O	-5.480186	0.408583	-0.031344
Si	-6.655706	1.550944	-0.578350
C	-8.109939	0.600838	-1.282348
C	-7.090150	2.492444	0.971076
C	-5.856364	2.631637	-1.885312
C	-6.086094	-2.583934	1.145542
C	-3.247737	-3.579728	1.670260
H	6.230361	-2.580410	1.392611
H	6.853305	-0.958483	3.168449
H	5.719031	1.245224	3.343195
H	1.704603	3.856491	2.219008
H	0.534203	3.476061	0.929064
H	0.887116	2.276996	2.194336

H	4.267129	3.542470	-0.816057
H	2.646642	4.235222	-0.997810
H	3.615242	4.607216	0.456683
H	4.857380	-0.858115	-3.061935
H	3.405207	-1.436121	-3.894832
H	4.578116	-2.610531	-3.233748
H	2.761989	-3.996846	-1.796035
H	1.403947	-2.998112	-2.376338
H	1.634677	-3.264345	-0.632122
H	1.002080	0.504485	-1.221613
H	2.280272	1.095981	-2.057508
H	-0.678099	-1.041828	-2.258978
H	-2.367843	-0.982860	-2.756522
H	-1.742465	-2.415420	-1.921547
H	-2.780246	1.706946	-1.129641
H	-1.131187	1.688712	-0.506159
H	-2.499808	1.710737	0.619603
H	0.086873	-0.719234	0.779737
H	-1.158798	-0.813673	2.029575
H	-0.770449	-2.237528	1.036766
H	-4.280905	-0.105231	-1.625617
H	-8.889598	1.297676	-1.605765
H	-7.836853	0.002344	-2.156824
H	-8.559788	-0.065721	-0.541226
H	-6.579950	3.367888	-2.249080
H	-5.002468	3.185961	-1.486648
H	-5.520283	2.063922	-2.758468
H	-7.830867	3.267421	0.752786
H	-7.517738	1.835317	1.732861
H	-6.213170	2.984865	1.399004
H	-4.619771	-1.428221	3.902696
H	-3.216893	-0.600922	3.234139
H	-4.848757	-0.027676	2.846414
H	-3.776742	-4.318966	2.281599
H	-3.074741	-4.040995	0.694289
H	-2.285680	-3.396459	2.147616
H	-6.466245	-3.138403	2.010632
H	-6.756902	-1.740677	0.980016
H	-6.140664	-3.261410	0.289425
H	-5.428648	-1.362276	-1.078178

IM12b

C	1.363447	-0.643318	0.348214
C	-0.011053	-0.864493	0.472804
C	-0.876808	0.108520	-0.034747
C	-0.410198	1.280098	-0.614871
C	0.970911	1.465766	-0.698236
C	1.874819	0.513080	-0.224578
Ir	-0.738641	-2.565625	1.382237
P	-2.749893	-1.554111	0.705620
C	-3.994880	-1.009475	1.915885
O	-2.247836	-0.108123	0.043752
O	2.242876	-1.615020	0.811918
P	1.572840	-3.005671	1.445067
C	2.509208	-3.187989	2.995799
C	-1.329898	-3.251475	3.301313
O	-0.957325	-1.938487	3.582942
Si	-0.752206	-1.037884	5.065009
C	0.245823	-2.101758	6.237400
C	0.129248	0.511011	4.521199
C	-2.460961	-0.708229	5.751128
C	2.219047	-4.310770	0.354126
C	-3.701347	-2.317534	-0.643417
H	1.352740	2.372826	-1.151382
H	-1.103520	2.019371	-0.993747
H	2.943805	0.661115	-0.302620
H	3.580005	-3.179911	2.782367
H	2.245113	-4.132343	3.476953
H	2.273682	-2.363497	3.667385
H	-4.717796	-0.348039	1.434295
H	-4.516815	-1.880940	2.317932
H	-3.505968	-0.479437	2.732077
H	1.802619	-4.187938	-0.645820
H	3.308096	-4.255431	0.302390
H	1.920431	-5.287289	0.742067
H	-4.474618	-1.631547	-0.994601
H	-4.165859	-3.238124	-0.282979
H	-3.031651	-2.561138	-1.468400
H	-0.666292	-3.115153	-0.069935
H	-1.237215	-4.094864	1.307952
H	-2.376598	-0.194059	6.713979
H	-3.055409	-0.068080	5.094584
H	-3.019316	-1.631765	5.928076
H	0.274932	1.178647	5.375989
H	1.114007	0.297495	4.098467
H	-0.445592	1.058312	3.769954
H	0.372693	-1.572542	7.187313
H	-0.255055	-3.047297	6.463567
H	1.245331	-2.326775	5.858080
H	-0.693545	-4.001765	3.765131
H	-2.391989	-3.443821	3.434410

TS13b

C	-1.168755	0.287600	-0.384484
C	0.179714	0.652882	-0.337841
C	1.125150	-0.376096	-0.378466
C	0.763354	-1.715190	-0.429038
C	-0.595910	-2.029034	-0.454563
C	-1.577728	-1.037792	-0.436325
Ir	0.748923	2.618522	-0.214410
P	2.849130	1.577346	-0.367396
C	4.072185	1.678485	0.977725
O	2.476454	-0.043380	-0.372486
O	-2.133136	1.290959	-0.383001
P	-1.582778	2.860633	-0.368954
C	-2.578976	3.589939	0.970525
C	1.282846	4.327220	1.047716
O	1.005843	3.341190	2.002706
Si	0.889654	3.448088	3.740734
C	-0.274750	4.856689	4.146492
C	0.233344	1.775381	4.235156
C	2.611290	3.786738	4.390070
C	-2.277124	3.558760	-1.899440
C	3.812600	1.785878	-1.897186
H	-0.896594	-3.068939	-0.496485
H	1.522621	-2.485750	-0.453135
H	-2.630957	-1.283996	-0.466079
H	-3.640016	3.414851	0.781143
H	-2.395903	4.665672	1.022022
H	-2.305839	3.132568	1.921157
H	4.873010	0.956124	0.807119
H	4.497807	2.683959	1.013756
H	3.588022	1.460514	1.929049
H	-1.821859	3.067992	-2.759893
H	-3.358362	3.410407	-1.927054
H	-2.054195	4.627249	-1.946273
H	4.649081	1.084540	-1.916024
H	4.193052	2.808497	-1.952321
H	3.168407	1.604753	-2.757835
H	0.732334	2.567060	-1.763205

H	1.177876	4.175331	-0.556307
H	2.586488	3.863172	5.481805
H	3.311598	2.986901	4.136340
H	3.017317	4.729728	4.012784
H	0.146220	1.714235	5.324317
H	-0.757184	1.582507	3.815595
H	0.896922	0.968890	3.913207
H	-0.371388	4.947995	5.233122
H	0.093476	5.819076	3.779565
H	-1.278947	4.698325	3.745713
H	0.599523	5.173626	1.099069
H	2.324761	4.643008	1.055816

IM13b

C	-0.688875	-0.457027	0.488911
C	0.636124	-0.823594	0.239779
C	1.612912	0.175104	0.259603
C	1.286389	1.506316	0.481495
C	-0.048952	1.839822	0.703784
C	-1.046729	0.866200	0.713238
Ir	1.120807	-2.724653	-0.130885
P	3.267068	-1.793663	-0.134033
C	4.386116	-1.793383	-1.571542
O	2.941253	-0.171110	0.040601
O	-1.672857	-1.438409	0.505600
P	-1.152835	-3.011412	0.348318
C	-2.375687	-3.693364	-0.815709
C	-1.588353	-3.766293	1.947870
C	4.355015	-2.161311	1.279411
O	0.976268	-3.062778	-2.461283
Si	0.654386	-2.158795	-3.915472
C	2.017013	-2.565059	-5.138054
C	1.250928	-4.469852	-2.508494
C	-1.013192	-2.693236	-4.582810
C	0.665516	-0.368584	-3.398295
H	-0.315407	2.874584	0.879655
H	2.066894	2.256205	0.481662
H	-2.083768	1.116574	0.896020
H	-3.386142	-3.470795	-0.466237
H	-2.250865	-4.777229	-0.878396
H	-2.232383	-3.261704	-1.804907
H	5.204929	-1.090246	-1.405068
H	4.798263	-2.795522	-1.712243
H	3.841293	-1.503323	-2.468703
H	-1.012883	-3.288317	2.740765
H	-2.654877	-3.645633	2.148065
H	-1.341957	-4.830887	1.930343
H	5.217004	-1.491142	1.280497
H	4.701379	-3.195865	1.215859
H	3.796241	-2.036636	2.207009
H	1.319874	-2.848173	1.402032
H	0.398623	-5.031560	-2.899443
H	0.461867	0.257401	-4.273040
H	-0.099598	-0.146964	-2.651090
H	1.630189	-0.055628	-2.992344
H	-1.190351	-2.209652	-5.548901
H	-1.076306	-3.771709	-4.753089
H	-1.834944	-2.400413	-3.924375
H	1.890989	-1.952549	-6.036544
H	3.013414	-2.351808	-4.741292
H	1.997732	-3.608921	-5.462870
H	2.141606	-4.682139	-3.103787
H	1.443667	-4.826243	-1.481958

TS14b

Si	2.641658	-0.337354	-0.030239
Ir	-1.299701	-0.008744	-0.281021
P	-1.585922	2.281575	-0.270355
C	-0.676182	3.405147	0.851520
C	-3.262826	0.125457	0.317448
C	-4.041660	-1.022061	0.536626
C	-5.354422	-0.956710	0.984586
C	-5.909664	0.304212	1.210120
C	-5.187567	1.478936	0.991812
C	-3.878037	1.366809	0.543680
O	-3.478345	-2.261630	0.282199
P	-1.891067	-2.237802	-0.270322
C	-1.158435	-3.475272	0.862555
O	-3.150906	2.519129	0.295185
C	2.077216	-0.378590	1.753957
C	2.396220	-1.796662	-1.183469
C	2.718709	1.311551	-0.907364
O	4.527394	-0.642975	0.366781
C	4.795341	-2.000490	0.859459
C	-2.040698	-3.132196	-1.855301
C	-1.629516	3.185831	-1.856243
Si	5.974184	0.424055	0.193401
C	6.443825	0.364069	-1.612165
C	5.515193	2.117809	
0.831105			
C	7.304810	-0.316136	1.278824
H	0.354717	-0.122743	-0.416267
H	-0.938975	-0.034212	-1.787802
H	-1.120774	-3.066456	1.872853
H	-1.761682	-4.385433	0.867708
H	-2.589574	-2.511252	-2.563465
H	-2.570437	-4.075948	-1.711941
H	-1.045158	-3.328416	-2.259329
H	-0.144103	-3.715312	0.536826
H	-1.156454	4.385462	0.876501
H	-0.666251	2.985458	1.857988
H	0.352603	3.513849	0.501504
H	-2.025741	4.192540	-1.709868
H	-2.265285	2.643234	-2.556064
H	-0.621515	3.246210	-2.272516
H	-5.630462	2.452424	1.156914
H	-6.933263	0.373178	1.559710
H	-5.926218	-1.861455	1.144567
H	3.331874	-2.080166	-1.673204
H	1.994613	-2.673545	-0.671520
H	1.678218	-1.515011	-1.953663
H	3.585725	1.400468	-1.564513
H	1.824961	1.390375	-1.526669
H	2.718087	2.150390	-0.210015
H	2.672224	0.314122	2.355921
H	1.028662	-0.088967	1.817978
H	2.175004	-1.374810	2.192788
H	6.445936	2.660228	1.028539
H	4.935036	2.716138	0.129659
H	4.972409	2.063866	1.778625
H	7.357928	0.946671	-1.766320
H	6.653793	-0.658195	-1.938773
H	5.678646	0.779008	-2.271328
H	8.181782	0.336012	1.204415
H	7.022097	-0.345788	2.334326
H	7.626575	-1.314466	0.976319
H	5.174047	-1.948543	1.877714
H	3.866555	-2.562688	0.845850
H	5.515420	-2.489770	0.204837

TS14b-syn

Si	2.511084	1.026346	1.315642
Ir	-0.776084	0.121791	0.267764
P	-0.776046	-2.148286	0.781682
C	-0.779770	-2.675765	2.527086
C	-2.654719	-0.391834	-0.333605
C	-3.108238	-1.719353	-0.277637
C	-4.361142	-2.102314	-0.735450
C	-5.202668	-1.119576	-1.257151
C	-4.812206	0.218340	-1.318883
C	-3.550837	0.557300	-0.850071
O	-2.271727	-2.682310	0.263973
O	-3.159498	1.886443	-0.884874
P	-1.650850	2.223144	-0.251686
C	-2.071526	3.367687	1.105456
C	1.695849	2.682986	1.714529
C	4.346693	1.512745	1.314586
O	2.884740	0.383315	-0.705993
C	2.203810	1.173884	-1.712688
C	2.337882	-0.322007	2.615356
C	-0.998141	3.310505	-1.567622
C	0.299843	-3.365324	-0.054477
Si	4.038643	-0.802315	-1.327023
C	3.175370	-1.692615	-2.732519
C	5.550692	0.117124	-1.930876
C	4.434208	-1.995532	0.056055
H	-6.184508	-1.401180	-1.618384
H	-5.469671	0.982082	-1.713165
H	-4.668345	-3.138317	-0.677948
H	-0.051762	-4.377242	0.157151
H	1.326792	-3.258360	0.298230
H	0.271060	-3.198377	-1.131802
H	-1.725168	4.093744	-1.791799
H	-0.063634	3.772085	-1.242914
H	-0.819075	2.727449	-2.471630
H	-1.502137	-2.076452	3.081859
H	-1.058204	-3.729207	2.594886
H	0.209951	-2.530290	2.962810
H	-2.724231	4.158495	0.730532
H	-1.165614	3.810575	1.522119
H	-2.590256	2.816106	1.890029
H	-0.992454	0.430527	1.760383
H	0.920391	0.513302	0.591937
H	0.709056	2.529674	2.150811
H	2.325639	3.235320	2.418491
H	3.087569	-0.176569	3.398930
H	1.585967	3.311699	0.826391
H	1.342832	-0.290170	3.061527
H	4.559316	1.943269	2.300018
H	4.575188	2.281658	0.571852
H	5.044753	0.687034	1.172405
H	2.493400	-1.318107	2.196851
H	5.142477	-2.733893	-0.334699
H	4.903087	-1.530941	0.924147
H	3.554119	-2.546735	0.393539
H	3.817351	-2.506709	-3.084218
H	2.229061	-2.139218	-2.417212
H	2.978045	-1.050009	-3.593648
H	6.255819	-0.597599	-2.367749
H	5.316230	0.848214	-2.709373
H	6.067834	0.638893	-1.123235
H	2.850695	1.313254	-2.580039
H	1.280650	0.678295	-2.014907
H	1.974757	2.153420	-1.297999

IM14b

C	4.063955	-1.624466	2.424574
C	3.224008	-1.439440	1.332829
C	2.854065	-0.163655	0.880922
C	3.389693	0.942450	1.558197
C	4.232676	0.802958	2.654485
C	4.554338	-0.489524	3.073514
Ir	1.576289	0.076824	-0.722309
P	1.800123	-2.209774	-0.702245
C	0.442808	-3.407345	-0.420790
O	2.739417	-2.546867	0.660219
O	3.071181	2.211837	
1.110458			
P	2.110757	2.267684	-0.277085
C	3.209984	3.136590	-1.449654
C	0.925529	3.564650	0.238012
C	2.783357	-2.991887	-2.029084
C	-2.130301	0.004374	0.898858
Si	-3.020736	0.266453	-0.708351
O	-4.842084	0.272633	-0.335847
Si	-5.707119	-0.197269	1.211838
C	-5.018917	0.876665	2.569926
C	-2.704006	1.953421	-1.432169
C	-2.819728	-1.149030	-1.902775
C	-5.688776	0.477912	-1.528905
C	-7.509627	0.186446	0.916795
C	-5.394323	-2.024912	1.403156
H	4.333544	-2.620683	2.750358
H	5.212985	-0.615950	3.925296
H	4.631874	1.674163	3.157358
H	1.460640	4.440225	0.611385
H	0.310430	3.853348	-0.617130
H	0.282626	3.175007	1.027660
H	4.036792	2.476639	-1.712040
H	2.652376	3.373046	-2.358542
H	3.599454	4.054811	-1.005696
H	3.699438	-2.419240	-2.173474
H	2.211862	-2.972996	-2.959688
H	3.032561	-4.022629	-1.769329
H	0.849532	-4.402191	-0.227870
H	-0.198383	-3.442733	-1.304245
H	-0.146785	-3.090110	0.439783
H	0.170063	0.256288	-1.510542
H	1.609498	0.223094	-2.291065
H	-1.757074	-1.204700	-2.159709
H	-3.374355	-1.022279	-2.835084
H	-3.107209	-2.104204	-1.455716
H	-3.181224	2.125325	-2.398935
H	-1.622863	2.029733	-1.587645
H	-3.003063	2.752626	-0.749083
H	-1.066858	-0.057391	0.620282
H	-2.235596	0.842991	1.588154
H	-2.386903	-0.921897	1.415634
H	-5.712723	0.827274	3.416239
H	-4.042182	0.558218	2.932680
H	-4.957252	1.925939	2.269084
H	-5.915970	-2.385449	2.295879
H	-5.779251	-2.593334	0.552204
H	-4.337002	-2.266299	1.527481
H	-8.045289	-0.091687	1.831375
H	-7.699502	1.250165	0.753083
H	-7.962580	-0.381273	0.101809
H	-6.201101	-0.451868	-1.768815
H	-6.396629	1.275475	-1.322104
H	-5.047873	0.767759	-2.356063

TS15b

Ir	1.348139	0.007781	-0.031360
Si	-4.682388	-0.241389	-1.554817

O	-4.011463	-0.088650	0.070037
C	-2.211663	-0.025470	0.060675
C	3.395045	-0.011765	0.085448
C	4.140575	1.176155	0.069047
C	5.523483	1.189063	0.192167
C	6.184227	-0.033444	0.324576
C	5.492721	-1.246060	0.330339
C	4.110404	-1.212278	0.204813
P	1.740900	-2.269336	0.045495
C	1.407767	-3.315983	-1.411721
P	1.798606	2.268030	-0.218014
C	1.492531	3.139410	-1.791346
O	3.465461	2.378960	-0.088839
O	3.404821	-2.407808	0.184445
C	-4.014422	-1.844637	-2.253791
C	-4.100101	1.264059	-2.502167
C	-6.548203	-0.299182	-1.457983
C	1.200285	-3.290335	1.463129
C	1.281961	3.460781	1.067985
Si	-4.788852	0.244814	1.623294
C	-6.079907	-1.071020	1.935107
C	-3.456285	0.139090	2.935379
C	-5.494321	1.974870	1.523306
H	7.263477	-0.041560	0.421486
H	6.066056	2.125363	0.178458
H	6.011498	-2.191249	0.423713
H	1.695493	-4.263322	1.441994
H	0.118076	-3.431940	1.419922
H	1.452146	-2.781433	2.394429
H	1.813911	4.406502	0.945699
H	0.206469	3.635935	0.993050
H	1.503779	3.050372	2.053899
H	1.801142	-2.825480	-2.302336
H	1.884992	-4.290904	-1.294580
H	0.330067	-3.449509	-1.528126
H	1.985612	4.113463	-1.789742
H	0.417901	3.274246	-1.932007
H	1.882924	2.538151	-2.612681
H	1.001898	-0.074524	-1.531067
H	-0.348449	0.041124	0.069392
H	-3.011922	1.326370	-2.583000
H	-4.496630	1.231151	-3.521845
H	-4.485464	-2.037516	-3.223053
H	-4.459904	2.189143	-2.043295
H	-2.934759	-1.837241	-2.422025
H	-6.926851	-0.292248	-2.486015
H	-6.992423	0.560734	-0.952780
H	-6.917463	-1.211908	-0.986946
H	-4.251931	-2.691206	-1.603698
H	-6.389198	-1.011096	2.983911
H	-6.977967	-0.956084	1.327357
H	-5.679973	-2.076073	1.774544
H	-3.925712	0.362480	3.899283
H	-3.025803	-0.862364	3.020277
H	-2.642611	0.855889	2.805054
H	-5.949055	2.241134	2.482827
H	-4.717952	2.716514	1.315192
H	-6.269849	2.074925	0.760541
H	-2.084820	1.016130	0.299072
H	-2.028482	-0.754658	0.828562
H	-2.035298	-0.326300	-0.956588

IM15

Ir	1.304264	0.047358	-0.849657
Si	-5.063374	0.034072	-0.303020
O	-3.525539	-0.276157	0.307866
C	-0.076894	-0.656817	-3.194773
C	2.119014	0.506350	0.921669
C	1.851181	1.729970	1.545052
C	2.385863	2.055178	2.784252
C	3.223377	1.133072	3.410718
C	3.529696	-0.090607	2.816837
C	2.977231	-0.382515	1.577064
P	2.654745	-1.852619	-0.545765
C	4.135952	-2.007331	-1.594462
P	0.510431	2.244656	-0.623854
C	1.271339	3.525226	-1.672025
O	1.020201	2.644933	0.907518
O	3.281243	-1.597554	0.973309
C	-5.674316	-1.491251	-1.213338
C	-4.888234	1.477418	-1.492398
C	-6.213190	0.473693	1.114334
C	2.013274	-3.552312	-0.414662
C	-1.254956	2.680815	-0.575175
Si	-2.596633	-1.097069	1.405716
C	-3.306239	-2.788897	1.798433
C	-0.899726	-1.344429	0.564890
C	-2.371963	-0.073808	2.959632
H	3.649120	1.374610	4.376806
H	2.151943	3.009421	3.237922
H	4.185573	-0.805778	3.295947
H	2.794289	-4.212916	-0.032393
H	1.701944	-3.899445	-1.402861
H	1.156404	-3.580920	0.257057
H	-1.373080	3.677643	-0.144378
H	-1.649988	2.682281	-1.593859
H	-1.820329	1.956462	0.011052
H	4.672745	-1.058531	-1.606679
H	4.792566	-2.790625	-1.210556
H	3.836893	-2.255286	-2.615920
H	0.966587	4.518716	-1.337054
H	0.959503	3.381620	-2.709301
H	2.356940	3.441562	-1.616537
H	2.520919	0.675765	-1.569559
H	0.905176	-0.264295	-2.820822
H	-4.193061	1.242021	-2.303915
H	-5.852286	1.721652	-1.948692
H	-6.656299	-1.307493	-1.660326
H	-4.530245	2.376973	-0.983646
H	-4.994162	-1.774610	-2.022141
H	-7.213742	0.713804	0.741774
H	-5.852205	1.343841	1.670255
H	-6.320878	-0.354802	1.820842
H	-5.776934	-2.350775	-0.544713
H	-2.649317	-3.343285	2.475883
H	-4.278929	-2.702767	2.291809
H	-3.442938	-3.392013	0.896554
H	-0.158337	-1.686256	1.288226
H	-0.972945	-2.061794	-0.254148
H	-0.608057	-0.347205	0.171432
H	-1.722597	-0.581320	3.679462
H	-1.932009	0.905353	2.749651
H	-3.334173	0.097679	3.450828
H	-0.421329	0.074700	-3.924669
H	-0.836398	-0.794086	-2.428955
H	0.138075	-1.607408	-3.680042

(POCOP)IrH$_2$

C	-1.273848	1.812202	0.128891
C	-1.332481	3.206133	0.069575

C	-0.141620	3.915769	0.195885
C	1.089201	3.272631	0.377578
C	1.096122	1.873092	0.430488
C	-0.071506	1.125115	0.308570
O	-0.168953	5.298113	0.141101
P	1.340842	6.057390	0.306117
C	0.972822	7.225235	1.665507
Ir	2.842752	4.356332	0.561724
P	3.659064	2.231576	0.745460
C	4.375112	1.610396	2.310276
O	2.302484	1.219440	0.609512
C	1.337538	7.119354	-1.183661
C	4.740042	1.504080	-0.538849
H	1.443078	6.489676	-2.067239
H	0.411977	7.695164	-1.250708
H	0.845801	6.662564	2.590504
H	0.066108	7.796423	1.454957
H	1.819037	7.905214	1.785938
H	2.192572	7.797308	-1.136083
H	4.819185	0.421845	-0.415486
H	4.325653	1.732373	-1.520943
H	5.730617	1.958357	-0.465623
H	4.473435	0.522901	2.290202
H	3.728438	1.905388	3.136802
H	5.356013	2.068101	2.456202
H	3.913694	5.109656	1.471113
H	-0.041335	0.043822	0.352620
H	-2.192824	1.244247	0.032163
H	-2.272125	3.725347	-0.070182
H	4.115098	5.051168	-0.101550

(POCOP)IrH$_2$+CO$_2$

C	-1.290618	1.959439	0.517948
C	-1.305740	3.331923	0.284796
C	-0.135173	4.102050	0.315814
C	1.074601	3.446916	0.583204
C	1.132921	2.076531	0.821754
C	-0.061259	1.353560	0.785556
O	-2.508149	3.958384	0.015383
P	-2.402795	5.625231	-0.293967
C	-3.149494	5.700602	-1.963570
Ir	-0.190643	6.139783	-0.027171
P	2.057706	5.840607	0.264823
C	3.180832	6.006054	-1.171169
O	2.241291	4.187877	0.610785
C	-3.729833	6.247036	0.796945
C	3.011202	6.572647	1.640567
O	0.492668	5.806163	3.985871
C	-0.654775	5.747133	3.823724
O	-1.806973	5.689426	3.699260
H	-2.469933	5.231309	-2.675074
H	-4.114445	5.189510	-1.983719
H	-3.414247	6.146146	1.834499
H	-4.655483	5.690513	0.635244
H	-3.892898	7.305201	0.579855
H	-3.279083	6.747612	-2.246060
H	4.165196	5.589079	-0.947772
H	2.744317	5.482883	-2.022058
H	3.275878	7.063902	-1.425641
H	3.996432	6.107866	1.718036
H	2.461323	6.430697	2.569958
H	3.122091	7.643860	1.457176
H	-0.333263	7.620783	0.544001
H	2.079105	1.592529	1.027792
H	-0.032733	0.285043	0.969766
H	-2.208046	1.385380	0.490367
H	-0.130217	7.405300	-1.001450

TS17

C	-1.212448	1.745961	-0.021097
C	-1.258668	3.134104	0.047086
C	-0.118791	3.914628	0.238467
C	1.099303	3.243418	0.342920
C	1.197947	1.857698	0.281314
C	0.028109	1.118895	0.100603
O	-2.487526	3.779344	-0.089631
P	-2.450421	5.450042	0.015531
C	-3.315614	5.937358	-1.515094
Ir	-0.228363	5.960478	0.356148
P	2.046078	5.658647	0.578310
C	3.212188	6.239032	-0.699320
O	2.259858	3.999467	0.505987
C	-3.666431	5.764134	1.341465
C	2.866291	6.068161	2.157858
O	-0.608632	8.960551	2.301288
C	-0.532460	7.795861	2.109696
O	-0.544546	6.680056	2.607628
H	-2.695569	5.672234	-2.371609
H	-4.281076	5.432825	-1.591649
H	-3.239004	5.453918	2.295428
H	-4.590793	5.213907	1.154098
H	-3.879618	6.834594	1.389144
H	-3.464081	7.019440	-1.512526
H	4.208803	5.825768	-0.530495
H	2.848092	5.927200	-1.678382
H	3.256125	7.330077	-0.673884
H	3.854368	5.606249	2.209310
H	2.248195	5.710364	2.981821
H	2.963326	7.153011	2.243206
H	-0.336137	7.685202	0.570470
H	2.163429	1.375918	0.371135
H	0.084386	0.037611	0.051565
H	-2.122257	1.177249	-0.166531
H	-0.034297	5.945574	-1.184788

IM17

C	-1.210442	1.745474	-0.028741
C	-1.257639	3.133565	0.030841
C	-0.115854	3.914817	0.215338
C	1.102582	3.243014	0.325656
C	1.198154	1.857170	0.272094
C	0.028876	1.117425	0.095210
O	-2.487058	3.775254	-0.106243
P	-2.456235	5.442705	0.009827
C	-3.351951	5.938400	-1.499414
Ir	-0.223532	5.940080	0.325677
P	2.054074	5.651850	0.573319
C	3.243889	6.244403	-0.675306
O	2.264289	3.995578	0.487188
C	-3.630727	5.758987	1.369092
C	2.825036	6.058190	2.175692
O	-0.605812	8.978304	2.255040
C	-0.515458	7.821486	1.963035
O	-0.531783	6.702755	2.511611
H	-2.748892	5.680374	-2.370070
H	-4.318082	5.432828	-1.559266

H	-3.171366	5.451285	2.308750
H	-4.562750	5.212728	1.210519
H	-3.836744	6.830594	1.423945
H	-3.503779	7.020061	-1.486432
H	4.237072	5.829680	-0.490406
H	2.899156	5.942521	-1.664410
H	3.288655	7.335176	-0.637647
H	3.813214	5.600913	2.256998
H	2.179020	5.699191	2.977214
H	2.912793	7.143405	2.267338
H	-0.347299	7.731962	0.648859
H	2.163946	1.377068	0.366640
H	0.084321	0.035976	0.052828
H	-2.121999	1.178311	-0.168669
H	-0.027908	5.887446	-1.220635

TS18

C	-1.217372	1.743254	0.040768
C	-1.270506	3.131510	0.004949
C	-0.120791	3.915067	0.132825
C	1.102821	3.258379	0.284508
C	1.189916	1.872081	0.324797
C	0.020725	1.122804	0.201077
O	-2.498264	3.758425	-0.168087
P	-2.477637	5.428777	-0.125903
C	-3.434829	5.843709	-1.621560
Ir	-0.224890	5.903396	0.113165
P	2.051773	5.671103	0.453298
C	3.306628	6.245426	-0.737902
O	2.263686	4.014811	0.392294
C	-3.574621	5.819875	1.271725
C	2.722971	6.095547	2.092657
O	-0.640333	8.551519	3.282989
C	-0.412737	7.874818	2.304758
O	-0.591395	6.598050	2.166747
H	-2.867061	5.542099	-2.501905
H	-4.400446	5.333449	-1.613888
H	-3.029101	5.630720	2.196623
H	-4.489450	5.225669	1.226525
H	-3.823581	6.883658	1.243017
H	-3.596004	6.923688	-1.658954
H	4.284164	5.819590	-0.501682
H	3.007640	5.944483	-1.742013
H	3.367310	7.335668	-0.699140
H	3.704723	5.641263	2.240412
H	2.022168	5.745033	2.850900
H	2.802348	7.181580	2.182924
H	0.002100	8.379277	1.377910
H	2.156433	1.401185	0.452334
H	0.074815	0.041228	0.232960
H	-2.131969	1.171656	-0.053851
H	-0.009466	5.660179	-1.429812

IM18

C	-1.189857	1.639904	-0.149942
C	-1.272950	3.023622	-0.051148
C	-0.152083	3.827816	0.174011
C	1.081945	3.182158	0.277936
C	1.213131	1.801784	0.184403
C	0.063971	1.039412	-0.029429
O	-2.519945	3.638301	-0.188054
P	-2.540539	5.296401	0.009735
C	-3.442074	5.858072	-1.471558
Ir	-0.321059	5.836909	0.369827
P	1.955811	5.607820	0.672045
C	3.157316	6.307881	-0.510933
O	2.217942	3.963724	0.477410
C	-3.709264	5.552536	1.384545
C	2.721123	5.896168	
2.307034			
O	-0.382545	9.454979	2.478107
C	-0.066340	8.598582	1.690750
O	-0.845229	7.628608	1.260783
H	-2.830474	5.678210	-2.355855
H	-4.392918	5.329788	-1.569322
H	-3.319936	5.059860	2.276311
H	-4.699438	5.157177	1.149281
H	-3.765169	6.625187	1.584339
H	-3.625701	6.931335	-1.382776
H	4.148563	5.877752	-0.352684
H	2.824912	6.092533	-1.526641
H	3.202745	7.391191	-0.379516
H	3.733274	5.486840	2.335614
H	2.114073	5.412217	3.073416
H	2.750540	6.968268	2.513252
H	0.955801	8.578587	1.243050
H	2.188867	1.341716	0.276698
H	0.146671	-0.038538	-0.103714
H	-2.084720	1.053906	-0.317678
H	-0.046681	5.608271	-1.133824

IM19

C	0.291587	1.283588	-0.398011
C	-0.907653	1.896041	-0.033208
C	-1.082537	3.226088	-0.419123
C	-0.130126	3.920355	-1.156819
C	1.046277	3.262038	-1.513097
C	1.271376	1.938894	-1.135398
Ir	-2.309330	0.919498	1.049178
O	-3.226069	0.095774	-0.807164
C	-4.446922	-0.296191	-0.846819
O	-5.283958	-0.280696	0.056695
O	-2.246609	3.886053	-0.034094
P	-3.371757	2.951825	0.774907
C	-3.779679	4.023968	2.194509
O	0.521562	-0.027595	0.009748
P	-0.713607	-0.745012	0.877292
C	0.201603	-1.400507	2.315652
C	-4.826448	3.076354	-0.317178
C	-1.076426	-2.213357	-0.141567
Si	-4.453003	-0.659854	3.031517
C	-4.583194	-2.411618	2.378712
C	-3.566165	-0.613374	4.693784
C	-6.109129	0.212143	3.121555
H	-1.584609	-1.872601	-1.043521
H	-0.157222	-2.744739	-0.396403
H	0.528777	-0.566707	2.937044
H	1.069347	-1.977196	1.988230
H	-0.457968	-2.041580	2.905420
H	-1.748738	-2.879440	0.404062
H	-5.144454	4.115166	-0.426935
H	-4.554579	2.668999	-1.291217
H	-5.632761	2.464407	0.090085
H	-4.024566	5.033193	1.856519
H	-2.926360	4.058829	2.871660
H	-4.636524	3.602238	2.725339
H	-4.734289	-0.687534	-1.845486
H	-0.311657	4.950193	-1.437595
H	1.798755	3.788069	-2.088966
H	2.184640	1.420560	-1.399306
H	-3.575477	0.182636	2.105822

H	-5.194754	-3.015112	3.058005
H	-5.055735	-2.401251	1.396130
H	-3.605453	-2.894037	2.299155
H	-6.781948	-0.327918	3.795997
H	-6.008507	1.232049	3.502946
H	-6.556202	0.243583	2.127429
H	-4.145888	-1.150389	5.451943
H	-2.578627	-1.078385	4.636616
H	-3.429136	0.413914	5.041328
H	-1.613764	1.479168	2.351349

TS20

Ir	-0.093210	-0.071090	0.407128
H	-0.052966	0.365542	1.903028
P	2.214216	-0.030255	0.482193
C	3.278135	-0.932291	-0.701325
C	3.063424	-0.271505	2.082782
O	2.627705	1.544321	0.104798
C	1.560243	2.426077	-0.074939
C	1.831851	3.766064	-0.327742
C	0.755878	4.641122	-0.476174
C	-0.559143	4.190706	-0.359699
C	-0.774795	2.841119	-0.105552
C	0.265192	1.921698	0.023486
O	-2.083660	2.384831	0.046428
P	-2.252554	0.752569	0.354161
C	-3.242778	0.757761	1.889569
H	-2.972985	0.396141	-1.911346
O	-0.234987	-1.291668	-1.514864
C	-0.549054	-0.941661	-2.757622
O	-0.770574	0.187289	-3.110545
H	-0.594083	-1.800517	-3.448405
H	-0.459022	-1.725225	0.737202
Si	-0.290689	-2.972621	-0.534988
C	0.382170	-3.935792	0.983482
C	0.761425	-3.804414	-1.893759
C	-2.105789	-3.443427	-0.740616
C	-3.451137	0.279487	-0.938880
H	2.860658	-0.838538	-1.703823
H	4.286100	-0.512272	-0.687964
H	2.585965	0.361519	2.830862
H	4.121188	-0.011686	2.003753
H	2.961917	-1.313645	2.393931
H	3.323238	-1.989662	-0.431503
H	-4.334154	0.919970	-0.884927
H	-3.745727	-0.763169	-0.803414
H	-4.142889	1.364167	1.768986
H	-2.635802	1.158942	2.701129
H	-3.520961	-0.269129	2.137674
H	-1.399857	4.865293	-0.462276
H	0.944598	5.689314	-0.677355
H	2.856114	4.109017	-0.403502
H	0.812119	-4.881978	-1.709947
H	0.370005	-3.655356	-2.903248
H	1.789750	-3.429835	-1.889658
H	-2.182276	-4.535004	-0.781164
H	-2.708305	-3.103718	0.105184
H	-2.546847	-3.046399	-1.658025
H	0.399028	-5.012706	0.780048
H	1.406802	-3.630798	1.220487
H	-0.224338	-3.750307	1.873543

IM20

Ir	-0.132356	-0.057444	0.810342
H	-0.178392	0.524368	2.238023
P	2.154871	0.052113	0.988617
C	3.356275	-1.041586	0.138098
C	2.886870	0.214268	2.659373
O	2.600499	1.511062	0.280601
C	1.542205	2.343785	-0.094189
C	1.822673	3.623786	-0.562818
C	0.748638	4.450234	-0.897148
C	-0.570518	4.017139	-0.751536
C	-0.790651	2.727426	-0.278881
C	0.246323	1.852081	0.037433
O	-2.100991	2.290047	-0.089557
P	-2.266264	0.739644	0.534917
C	-3.296369	1.061193	2.012561
H	-2.973324	-0.033290	-1.633039
O	-0.126684	-1.523184	-1.391491
C	-0.148435	-0.870663	-2.583769
O	-0.610837	0.218289	-2.731517
H	0.317309	-1.445608	-3.399145
H	-0.450599	-1.524441	1.531356
Si	-0.289786	-3.253760	-1.194499
C	1.059828	-3.880712	-0.069565
C	-0.118319	-4.032747	-2.901187
C	-2.000981	-3.565153	-0.513270
C	-3.463978	0.046340	-0.663484
H	3.047466	-1.187599	-0.897202
H	4.347153	-0.582725	0.153388
H	2.349388	0.991910	3.202456
H	3.945179	0.476962	2.599048
H	2.767135	-0.729081	3.196604
H	3.392556	-2.009698	0.640016
H	-4.330409	0.705469	-0.752439
H	-3.786927	-0.942350	-0.332188
H	-4.177212	1.651404	1.750924
H	-2.700514	1.602519	2.747579
H	-3.604332	0.108291	2.448479
H	-1.405928	4.662607	-0.993384
H	0.942238	5.451510	-1.265526
H	2.846997	3.963590	-0.654975
H	-0.230988	-5.116937	-2.795910
H	-0.885804	-3.698173	-3.604690
H	0.861378	-3.856143	-3.354306
H	-2.153237	-4.638281	-0.358268
H	-2.121699	-3.056035	0.444453
H	-2.776072	-3.214058	-1.199802
H	0.907841	-4.950465	0.109817
H	2.050494	-3.756063	-0.512757
H	1.027427	-3.361562	0.889049
